# STAT3 Signaling Contributes to Chemokine Production in E2-Induced Dermal Inflammation

**DOI:** 10.3390/ijms27219454

**Published:** 2026-10-23

**Authors:** Nicolas Ancona, Yucui Li, Ludivine Renaud, Roselyn Fierkens, Kaelyn Kear, Kaitlyn Pinckney, DeAnna Baker Frost

**Affiliations:** 1 Department of Medicine, Division of Rheumatology and Immunology, Medical University of South Carolina, Charleston, SC 29425, USA; ancona@musc.edu (N.A.); lisensi@163.com (Y.L.); renaudl@musc.edu (L.R.); keark@musc.edu (K.K.); 2 Department of Pediatrics-Rheumatology, University of Colorado Anschutz Medical Campus, Aurora, CO 80045, USA; 3 Department of Biological & Physical Sciences, South Carolina State University, Orangeburg, SC 29117, USA

**Keywords:** systemic sclerosis, dermal inflammation, estrogen

## Abstract

Scleroderma (SSc) is characterized by dermal inflammation and fibrosis. Patients with SSc have elevated circulating levels of estradiol (E2) that contribute to dermal fibrosis. Using bulk RNA sequencing (RNA-seq), we investigated inflammatory pathways in E2 or ethanol (ETOH, vehicle)-treated human skin tissue for 48 h to identify differentially expressed genes (DEGs) and enriched pathways. Among the 90 DEGs responsive to E2 treatment, many were associated with enriched inflammatory pathways. Likewise, cytokine and chemokine activities were among the most significantly enriched molecular function terms. qPCR and protein analyses demonstrated increased cytokine and chemokine expression in primary dermal fibroblasts. CIBERSORTx deconvolution using a skin single-cell reference suggested an activated fibroblast transcriptional signature in E2-treated human skin tissue. In this tissue, E2-induced *IL-6* and *CXCL8* transcript levels were positively correlated. Pharmacologic STAT3 inhibition reduced E2-induced *CXCL2*, *CXCL3*, *CXCL8*, and *CCL20* transcript levels in human skin tissue. In SSc dermal fibroblasts, anastrozole treatment significantly decreased *CXCL3* and *CXCL8* transcript levels. Together, these data suggest that E2 contributes, in part, to increased *CXCL2*, *CXCL3*, *CXCL8*, and *CCL20* transcripts and that IL-6/STAT3 signaling may be important in this dermal inflammatory response.

## 1. Introduction

Systemic sclerosis (SSc) is an autoimmune disease with a female predominance, especially during childbearing years [1], suggesting a role for sex hormones in disease pathogenesis [2]. One hallmark of SSc is dermal fibrosis, characterized by increased extracellular matrix (ECM) synthesis [3]. Several factors contribute to dermal fibrosis, including estradiol (E2), the most abundant form of estrogen found in non-pregnant women [4]. Circulating E2 levels are elevated in patients with diffuse cutaneous (dc)SSc [5,6] and are associated with worse survival [6]. Mechanistically, E2 promotes fibrosis by increasing the ECM components collagen and fibronectin and the pro-fibrotic cytokine TGFβ in dermal fibroblasts [5,7].

Dermal inflammation is another hallmark of SSc [8,9] that precedes and perpetuates dermal fibrosis [10] partly through chronically active fibroblasts [11]. Primary human dermal fibroblasts derived from patients with SSc produce chemokines and cytokines in culture in the absence of additional stimulation [12]. E2 also helps create the inflammatory dermal microenvironment through a positive feedback loop with the pro-inflammatory cytokine IL-6, which perpetuates inflammation and contributes to ECM accumulation in the skin [13].

The feedback loop between E2 and IL-6 is intriguing since IL-6 is elevated systemically in patients with SSc [14] and is produced by SSc primary dermal fibroblasts [15]. Because IL-6 contributes significantly to disease progression in SSc, the IL-6 receptor inhibitor tocilizumab has received FDA approval for treating SSc-associated lung involvement [16]. Although tocilizumab is FDA-approved for SSc-associated interstitial lung disease, it is not approved for SSc-related skin involvement due to insufficient efficacy, underscoring the multifactorial nature of cutaneous disease in SSc. In addition to IL-6, E2 can stimulate the production of other cytokines [17,18], although these effects vary depending on cell type, E2 concentration, exposure duration, and whether the system is studied in vivo or in vitro [19].

Although E2 is known to participate in a positive feedback loop that may amplify dermal inflammation, it is unclear if additional inflammatory pathways are activated and which downstream signaling mechanism is important in this process. Here, we used bulk RNA sequencing (RNA-seq) of E2-treated human skin tissue to investigate E2-induced dermal inflammatory signatures, the probable cell type that contributes to the signature, and the role of STAT3 in E2-induced dermal inflammation.

## 2. Results

### 2.1. Perturbation of Cytokine/Chemokine and Inflammatory Pathways in E2-Treated Skin Tissues

**Figure 1:**
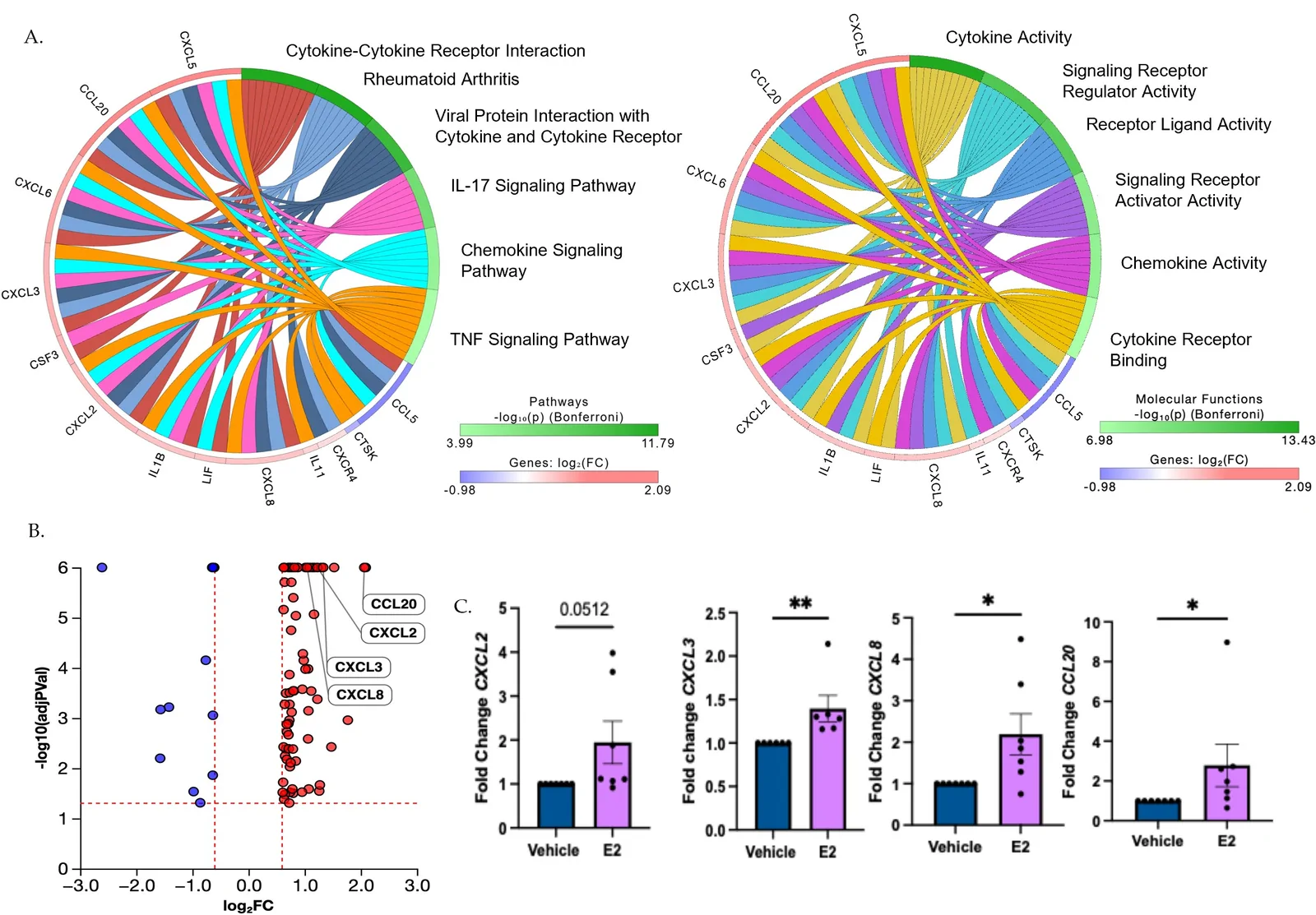
Perturbation of chemokine and inflammatory pathways in E2-treated human skin tissues. (**A**) Top six most impacted pathways from enrichment analysis and the top six impacted molecular functions of E2-stimulated human tissue (48 h) in organ culture. (**B**) Volcano plot highlighting the DEGs *CXCL2*, *CXCL3*, *CXCL8*, and *CCL20*. (**C**) Validation by qPCR of selected chemokine DEGs using human skin tissue RNA not analyzed by RNA-seq. The data is normalized to *beta-2 microglobulin* (*B2M*). n = 3 (**A**,**B**), n = 6–7 (**C**), Student’s *t*-test,* *p* < 0.05, ** *p* < 0.01.

Differential expression analysis “E2 vs. vehicle ethanol (ETOH)” in human skin tissue identified 90 differentially expressed genes (DEGs) with good clustering of the samples by treatment after batch effect correction (Figure S1, Table S1). The iPathwayGuide enrichment analysis revealed 51 impacted pathways and 1153 enriched gene ontology (GO) terms (Tables S2 and S3). The six most enriched pathways, including cytokine-cytokine receptor interaction and chemokine signaling, point to a strong inflammatory response in E2-treated human skin tissue (Figure 1A). Additionally, the top six most significant molecular function terms also pertained to inflammatory functions (Figure 1B): Cytokine, Receptor Ligand, Signaling Receptor Activator/Regulator, and Chemokine Activity. The genes for C-X-C motif chemokine ligand 2, 3, and 8 (*CXCL2*, *CXCL3*, and *CXCL8*) and *CCL20* were among the four most prominent DEGs contributing to this signature and were validated by qPCR in independent E2-treated human skin tissue that was not included in the bulk RNA-seq dataset (Figure 1B,C). Together these results suggest that while E2 induces fibrosis of skin tissues [7,20], it also promotes a noticeable inflammatory gene signature.

### 2.2. Dermal Fibroblasts Partially Mediate E2-Induced Cytokine/Chemokine Response in Skin Tissue

**Figure 2:**
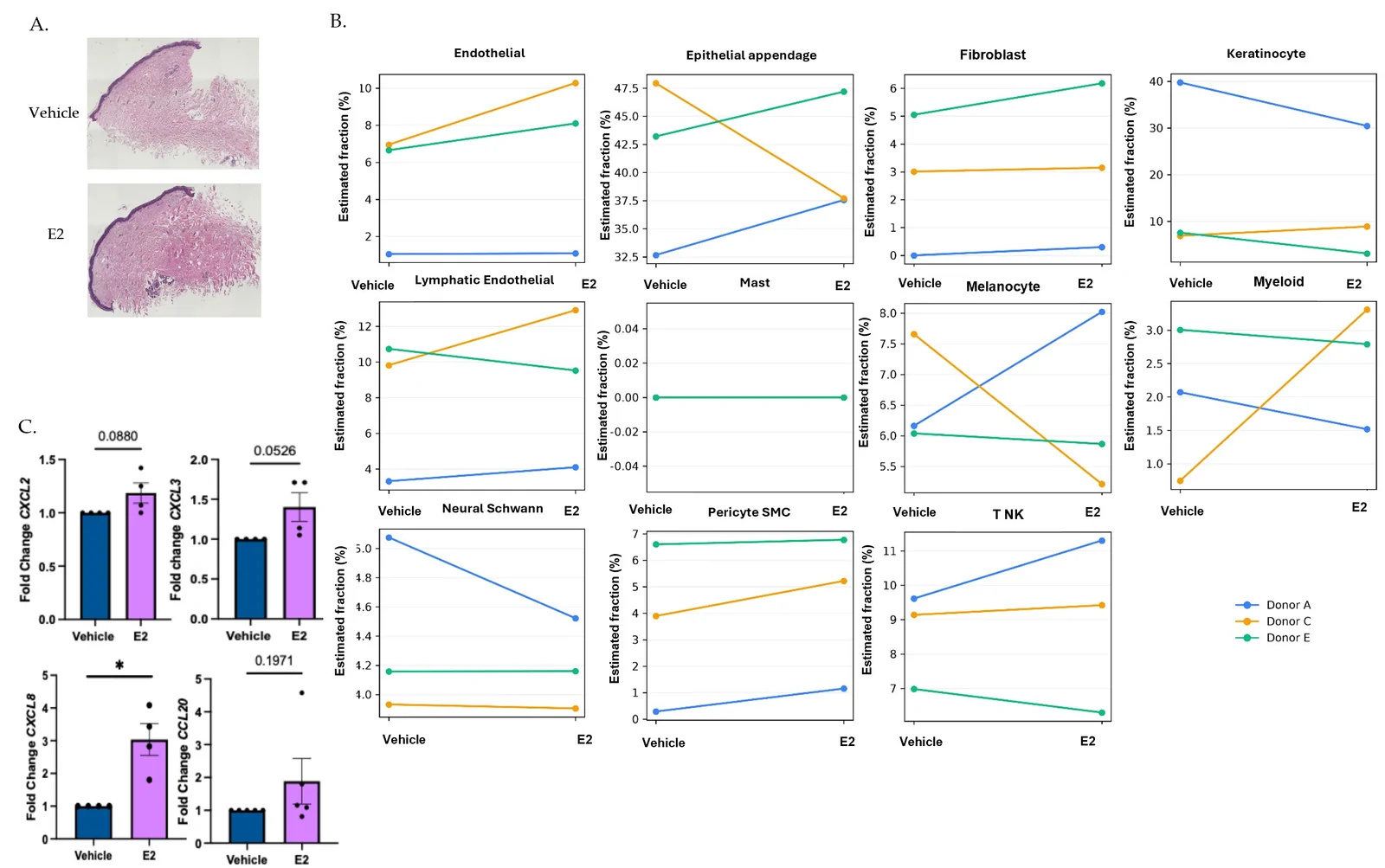
Cellular contribution of E2-induced chemokine/cytokine production in situ. (**A**) Vehicle (ETOH) and E2-treated hematoxylin and eosin (H and E)-stained human skin tissue sections. (**B**) CIBERSORTx deconvolution using a skin single-cell reference derived from GSE138669. Pericyte SMC = Pericyte smooth muscle cell, T/NK = T-/Natural Killer cells. (**C**) *CXCL2*, *CXCL3*, *CXCL8*, and *CCL20* steady-state transcript levels in vehicle or E2-treated dermal fibroblasts for 24- or 48 h normalized to *beta-2 microglobulin* (*B2M*), n = 4–5, Student’s *t*-test, * *p* < 0.05.

Figure 1 demonstrates enriched inflammatory pathways in E2-treated human skin tissue. Since our bulk RNA-seq data contains multiple dermal cell types, we asked which resident cells contribute to cytokine and chemokine production. We first considered if inflammatory cell infiltrates could account for this signature. A blinded dermal pathologist examined ETOH- and E2-treated, hematoxylin and eosin (H and E)-stained skin tissue sections (Figure 2A) and determined that E2-treated human skin tissues did not have a robust inflammatory infiltrate (Table S4). Therefore, the E2-induced transcriptional response is unlikely to be explained by the recruitment or expansion of immune cells during the 48 h culture.

Consistent with this observation, CIBERSORTx deconvolution of the bulk RNA-seq data revealed no consistent E2-associated increase in estimated myeloid or T-/Natural Killer (NK)-cell fractions across the three donor-matched samples (Figure 2B). Overall the cellular composition remained predominantly donor-dependent. Small directionally concordant increases were observed in fibroblast, endothelial, and pericyte/smooth-muscle cell (SMC) fractions, although these exploratory differences were not statistically significant. Together, these findings suggest that the E2-induced inflammatory transcriptional response primarily reflects responses within resident skin-cell populations rather than increased immune-cell abundance. We confirmed that dermal fibroblasts retain the ability to produce chemokines and cytokines in response to E2 treatment (Figure 2C). These findings suggest that because primary dermal fibroblasts can produce chemokines and cytokines in response to E2, they are a plausible resident effector population.

Because dermal fibroblasts are a possible resident effector population in our model, we next asked whether E2 changes which fibroblast states are present. Using CIBERSORTx, we performed an exploratory analysis using fibroblast-subtype signatures in our bulk RNA-seq data from GSE138669 [21,22]. Four fibroblast subtypes were based on eight markers (Table S5) [22,23]. Exploratory CIBERSORTx analysis of four fibroblast transcriptional states did not show a consistent change in total fibroblast abundance after E2 treatment, although an activated CTHRC1/COMP-associated signature emerged in two of three donor pairs (Figure S2). Given the small sample size and enrichment of this signature in SSc-derived tissue [22,23], this observation is considered hypothesis-generating but is consistent with E2 promoting an activated, disease-associated fibroblast transcriptional state.

### 2.3. IL-6 Correlates with E2-Induced Chemokine Responses in Human Skin Tissue

**Figure 3:**
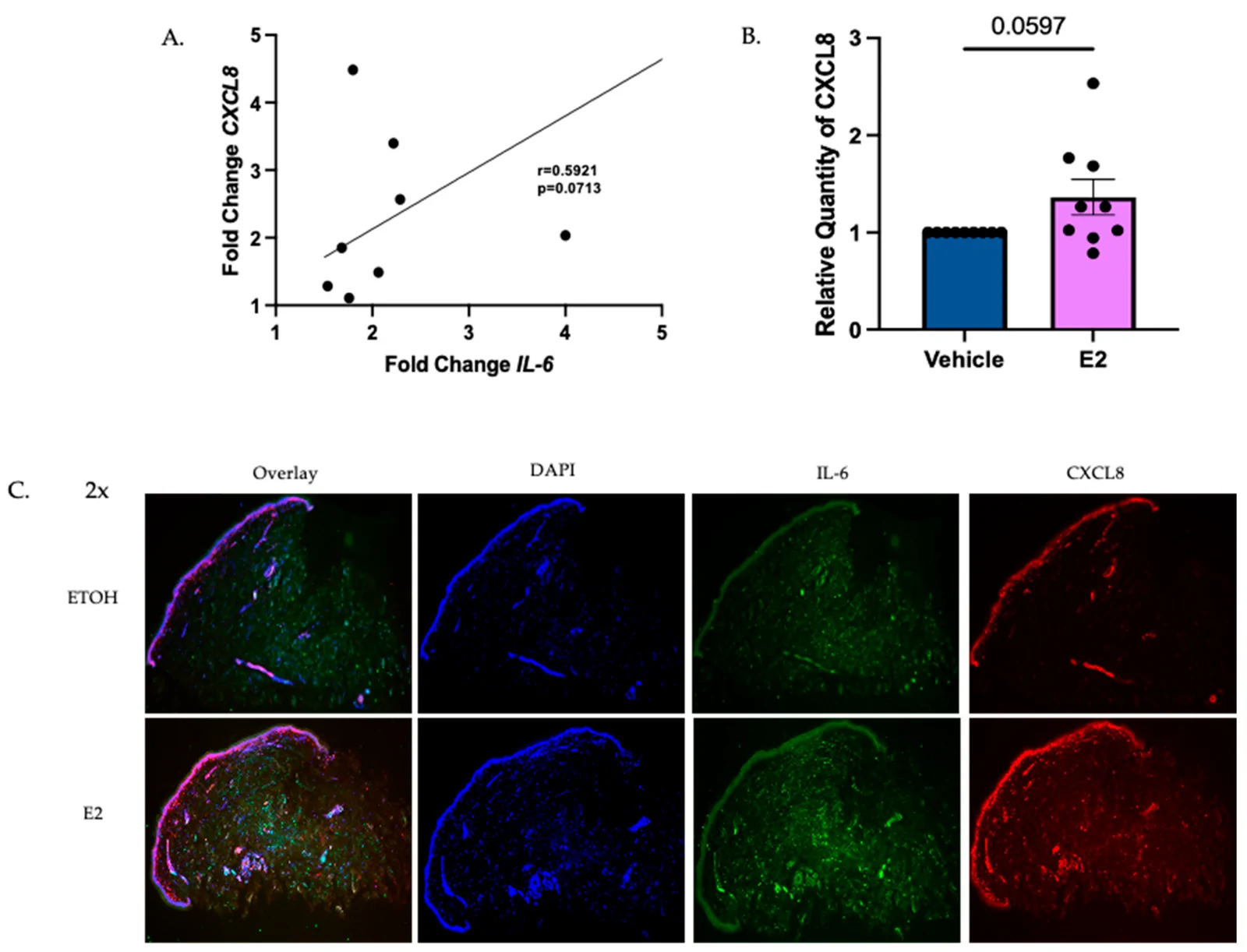
IL-6 correlates with E2-induced chemokine responses in human skin tissue. (**A**) Pearson’s correlation between *IL-6* and *CXCL8* steady-state transcript levels in human skin tissue treated with E2 or vehicle (ETOH) for 48 h in organ culture (n = 8). (**B**) Secreted CXCL8 protein levels from the conditioned media of E2-stimulated or vehicle-treated human skin tissue for 72 h measured by ELISA (n = 9). Statistical analysis: Student’s *t*-test. (**C**) Representative immunofluorescence of E2-stimulated or vehicle-treated human skin tissue in organ culture for IL-6, CXCL8, and DAPI.

We examined whether E2-induced chemokine production correlates with E2-driven IL-6 transcript levels. Given the positive feedback loop between E2 and IL-6 in human skin tissue [12] and the association of E2 with pro-fibrotic mediators [20], we examined whether E2-induced chemokine production correlates with E2-driven *IL-6* transcript levels. We found that *IL-6* and *CXCL8* transcript levels were significantly positively correlated in E2-stimulated human skin tissue in organ culture (Figure 3A).

We also measured CXCL8 protein by ELISA (secreted) and immunofluorescence (in situ). We detected an increase in CXCL8 protein in E2-stimulated human skin tissue, both secreted and in situ (Figure 3B,C). Notably, we detected an overlap in IL-6 and CXCL8 protein expression in tissue (Figure 3C Overlay).

### 2.4. STAT3 Blockade Prevents E2-Induced Chemokine Production in Human Skin Tissue

**Figure 4:**
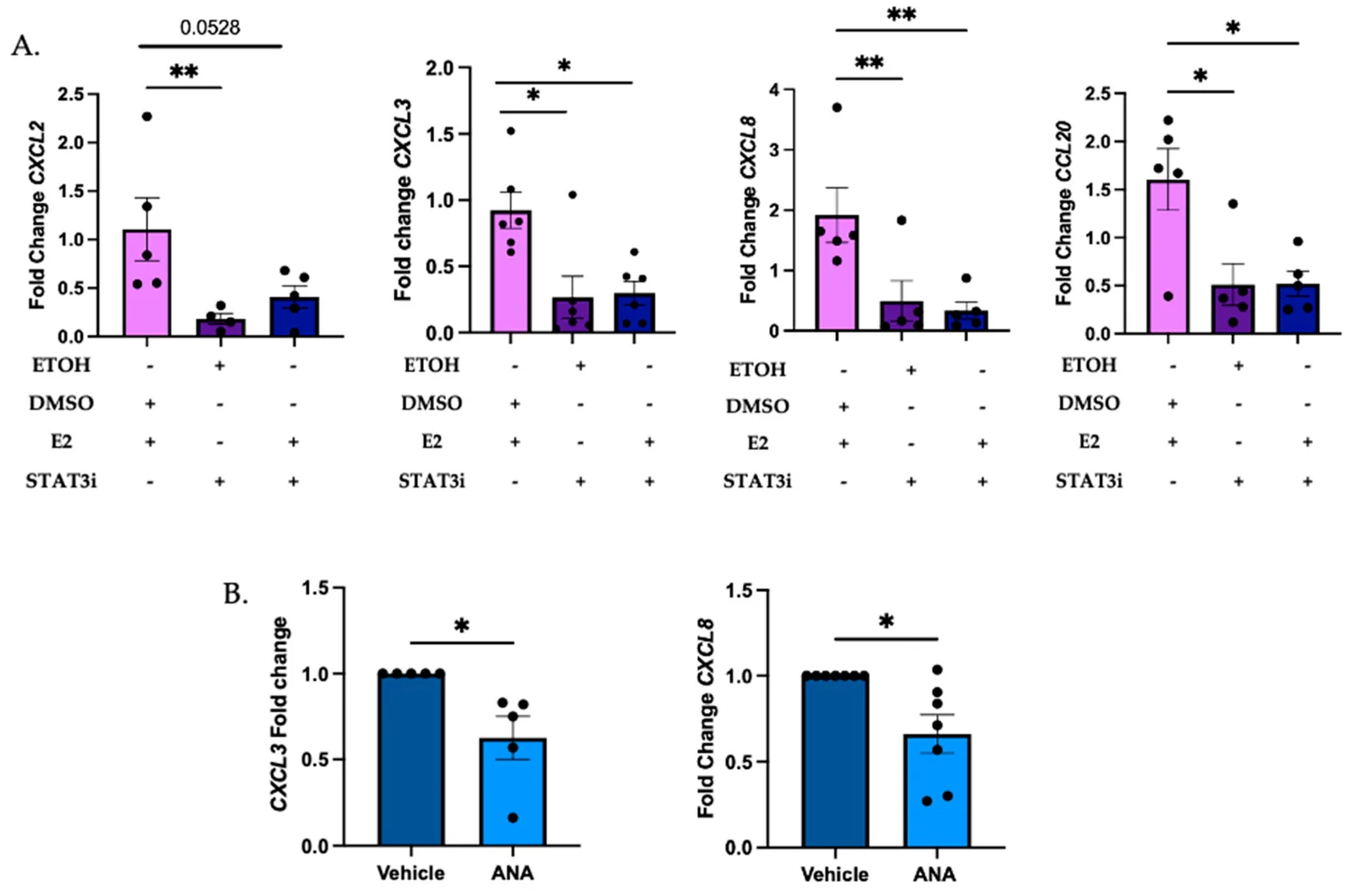
STAT3 inhibition and E2 production blockade in E2-induced chemokine production (**A**) Human skin tissue ex vivo was pretreated with a STAT3 inhibitor (STAT3i) or DMSO vehicle, followed by treatment with E2 or ethanol vehicle (ETOH) for an additional 72 h. Total RNA was isolated, and *CXCL2*, *CXCL3*, *CXCL8*, and *CCL20* steady-state transcript levels were measured, normalized to *beta-2 microglobulin* (*B2M*), relative to vehicle treatment (n = 5-7 individual samples). One-way, * *p* < 0.05, ** *p* < 0.01 relative to vehicle treatment. (**B**) Primary SSc dermal fibroblasts treated with 300 nM of anastrozole (ANA) to measure *CXCL3* and *CXCL8* steady-state transcript levels for 96 or 48 h, respectively. Normalized to *B2M*. n = 3-4 SSc donors, with 2–3 in duplicate. Student’s *t*-Test, * *p* < 0.05 relative to vehicle.

Based on our enrichment analysis, one of the six most impacted pathways in E2-stimulated human skin tissue was the IL-17 signaling pathway (Figure 1A). Additionally, E2 induces IL-6 production in human skin tissue [13]. Because an IL-17/IL-6/STAT3 relationship exists in the tumor microenvironment [24], we evaluated if E2-induced chemokine steady-state transcript levels depend upon STAT3. We found that E2-induced *CXCL2*, *CXCL3*, *CXCL8*, and *CCL20* steady-state levels were significantly reduced by pharmacologic STAT3 inhibition in human skin tissue (Figure 4A). This suggests that STAT3 is a common second messenger used by E2 to promote chemokine production in human skin tissue.

### 2.5. E2 Production Blockade Partially Inhibits E2-Induced Chemokine/Cytokine Production

In our previous work, genetic estrogen receptor alpha (ERα) deficiency in mice, the FDA-approved aromatase inhibitor, and the ERα inhibitor fulvestrant in SSc dermal fibroblasts decreased pro-fibrotic mediators [13]. These data suggest that E2 blockade may be a therapeutic target in SSc fibrosis. Therefore, we evaluated if blocking E2 production would have an impact on dermal inflammation. In primary human SSc dermal fibroblasts, aromatase inhibition with anastrozole (ANA) significantly decreased *CXCL8* and *CXCL3* steady-state transcript levels compared to vehicle ETOH after 48 and 96 h of treatment (Figure 4B), again demonstrating E2 mechanisms in SSc dermal inflammation.

## 3. Discussion

In this study, bulk RNA-seq identified 90 DEGs and strong enrichment of inflammatory pathways, including cytokine–cytokine receptor interaction, IL-17 signaling, chemokine signaling, and rheumatoid arthritis, supported by parallel enrichment of inflammatory GO molecular function terms. *CXCL2*, *CXCL3*, *CXCL8*, and *CCL20* emerged as dominant contributors to this signature. Since inflammatory infiltration was minimal, and primary dermal fibroblasts produced high levels of chemokines following E2 stimulation, our data indicates that resident dermal fibroblasts, rather than immune cells, contribute to the E2-induced inflammatory response, establishing them as effector cells. Additionally, E2-stimulated human skin tissue displayed a strong positive correlation and spatial co-expression of IL-6 and CXCL8, suggesting that IL-6 amplifies E2-driven inflammation through downstream chemokine induction. Our data suggests that STAT3 blockade reduced chemokine induction. Together, these findings suggest that E2 amplifies dermal inflammation, likely through IL-6/STAT3-dependent and parallel pathways. They also point to resident dermal fibroblasts as a likely source of E2-induced chemokines in human skin.

### 3.1. E2 Initiates Inflammation in Human Skin Tissue

Patients with SSc have abnormal systemic E2 levels [5,6], and elevated E2 levels are associated with worse mortality [6]. The impact analysis of bulk RNA-seq from E2-stimulated human skin tissue showed marked enrichment of inflammatory and chemokine signaling, a pattern that resembles the cytokine and chemokine expression reported in SSc patients [25,26]. This inflammatory signature was driven in part by the robust upregulation of several chemokines and cytokines, including *CXCL2*, *CXCL3*, *CXCL8*, *CCL20* and *IL-6*, a pattern that also closely mirrors the transcriptional profile observed in SSc dermal fibroblasts [25].

While E2 and IL-6 participate in a positive feedback loop [13], IL-6 exerts broader effects that perpetuate dermal inflammation. In our dataset, E2-stimulated human skin tissue showed that IL-17 signaling, which is known to induce IL-6 and CXCL8 production, was among the five most significantly enriched pathways in E2-treated human skin tissue [25]. Consistent with this, *IL-6* and *CXCL8* steady-state transcript levels were positively correlated and co-expressed within confined dermal regions. A similar positive correlation between IL-6 and CXCL8 has been reported in the serum of patients with acne vulgaris and is associated with disease severity [27]. In comparison, *CXCL8* showed a stronger association with *IL-6* than *CCL20*, although IL-6 signaling can directly impact CCL20 [28] and CXCL2 [29] production. Together, these findings suggest that IL-6 may further amplify dermal inflammation by promoting additional cytokine and chemokine production downstream of E2 stimulation.

### 3.2. STAT3 Inhibition Reduces E2-Induced Chemokine Expression in Human Skin Tissue

Dermal fibroblasts are the effector cells that produce pro-fibrotic mediators in response to E2 by activating the ERα signaling pathway [5,7,30,31]. However, due to the heterogeneity observed in SSc, we hypothesized that E2 is also implicated in additional pathogenic pathways that contribute to fibrosis.

The histopathology of E2-treated human skin tissue did not show a robust inflammatory infiltrate, and the transcriptomic deconvolution did not detect a consistent E2-associated increase in myeloid or T/NK-cell abundance. These findings argue against immune-cell accumulation as the principal explanation for the E2-induced inflammatory signature and instead implicate transcriptional activation of resident dermal cells. Because we saw little inflammatory infiltrate and E2 increased chemokine transcripts in primary dermal fibroblasts, fibroblasts are a likely source of the E2-induced signature. Other resident cells may also contribute, and deconvolution cannot establish that fibroblasts are the exclusive source. Dermal fibroblasts are increasingly recognized as active contributors to inflammation in autoimmune diseases, with distinct fibroblast subtypes, such as inflammatory fibroblasts identified in human skin [11,32,33]. Our primary dermal fibroblast experiments establish fibroblasts as one such E2-responsive effector population capable of producing IL-6 and chemokines [13]. Therefore, even in the absence of immune cells, E2 stimulation is sufficient to induce an inflammatory program in human dermal fibroblasts.

Our exploratory CIBERSORTx analysis demonstrated that E2 did not produce a consistent increase in overall fibroblast abundance. These findings raise the possibility of a shift toward a CTHRC1/COMP-associated activated fibroblast transcriptional state in responsive donors. CTHRC1/COMP is strongly SSc-enriched [22,34,35], so its appearance could reflect E2 induction of an SSc-like activation program—not necessarily expansion of a physically distinct fibroblast population.

### 3.3. ERα Signaling Inhibition Partially Impacts STAT3-Dependent Chemokine Production in Dermal Fibroblasts

Our data established the anti-fibrotic effects of ERα signaling inhibition and E2 production blockade in SSc dermal fibroblasts [5,7,13]. In this manuscript, we show a decline in *CXCL8* and *CXCL3* in active, primary SSc dermal fibroblasts, demonstrating a specific anti-inflammatory effect of the E2 production blocker anastrozole. This suggests that *CXCL8* and *CXCL3* are dependent, in part, on SSc dermal fibroblasts’ intrinsic ability to produce E2.

Even though anastrozole treatment significantly reduced the expression of select E2-induced chemokines in SSc dermal fibroblasts, not all chemokines were affected. This indicates that E2-driven chemokine production is not mediated solely through local E2 synthesis and suggests the involvement of additional signaling pathways. These findings imply that SSc fibroblasts retain alternative mechanisms capable of sustaining chemokine induction, even when E2 production is pharmacologically inhibited. While we describe that E2 and IL-6 participate in a positive feedback loop in dermal fibroblasts [13], our current data suggests that IL-6 has wider effects in perpetuating dermal inflammation. Hence, in the context of E2-treated dermal fibroblasts, IL-6 may further amplify dermal inflammation by promoting the expression of additional cytokines and chemokines.

### 3.4. Cross-Talk Between ERα and STAT3 as a Potential Driver of Dermal Pathogenesis

Our results suggest that STAT3 inhibition decreased E2-induced *CXCL2*, *CXCL3*, *CXCL8*, and *CCL20* steady-state transcript levels in human skin tissues. This is consistent with the established relationship between ERα and STAT3 observed in certain breast cancers, where JAK/STAT3 signaling can hijack ERα enhancers to promote metastasis [36]. Similarly, in breast, endometrial, and prostate malignancies, a cross-talk exists between ERα and STAT3 when activated through leptin signaling, leading to increased ERα-dependent cell viability [37]. Thus, the potential interaction between ERα and STAT3 provides a rationale for dual ERα and STAT3 inhibition as a potential treatment for dermal inflammation.

Several active pathways in SSc contribute to disease pathogenesis, including the PI3K/AKT [38], JAK/STAT [39], and MAPK/ERK [40], emphasizing the importance of selecting an antagonist capable of targeting multiple nodes within these pathways to achieve broad therapeutic benefit. While we previously demonstrated that E2 induces TGFβ through the MAPK/ERK pathway [7], the current study suggests that E2 also engages the JAK/STAT pathway to drive chemokine and cytokine production. This dual-pathway utilization amplifies dermal inflammation and directly shapes the inflammatory microenvironment.

### 3.5. Dual ERα/STAT3 Inhibition as a Potential Therapeutic Strategy for SSc Dermal Inflammation

Since our data showed that (1) dermal fibroblasts are effector cells of inflammation in E2-stimulated human skin tissues; (2) E2 inhibition with anastrozole significantly decreased *CXCL3* and *CXCL8* steady-state transcript levels in primary human SSc dermal fibroblasts; and (3) STAT3 inhibition decreased E2-induced *CXCL2*, *CXCL3*, *CXCL8*, and *CCL20* steady-state transcript levels in human skin tissue, it suggests that dual ERα/STAT3 inhibition could be a beneficial therapeutic strategy to counter SSc dermal inflammation.

Future directions include investigating selective estrogen receptor modulators (SERMs) as a potential treatment for E2-induced dermal inflammation. SERMs are used to treat hormonally responsive cancers and osteoporosis [38,39]. One known benefit of 3rd-generation SERMs is improved tissue specificity, which leads to fewer side effects [41]. In addition to modulating ER signaling, SERMs prevent STAT3 activation by binding to the IL-6 membrane receptor, gp130 [42]. Therefore, 3^rd^-generation SERMs can act as dual ERα and IL-6 receptor inhibitors and may be beneficial in diseases with a hormonal and IL-6/STAT3 component, such as SSc.

There is a lack of FDA-approved medications to specifically treat dermal inflammation or fibrosis. As such, non-specific immunotherapies are used to dampen inflammation [36], since uncontrolled inflammation leads to fibrosis [37]. Our data suggests E2 can function as a mediator between inflammation and fibrosis because the inflammatory cytokine and chemokine production in human skin tissue is dependent in part on the IL-6/JAK/STAT3 pathway. Thus, ERα and IL-6 signaling may be complementary, druggable targets for E2-induced dermal inflammation. Because E2-treated fibroblasts produce both pro-fibrotic mediators [5,7,30,31] and chemokines and cytokines [13], blocking ERα signaling with SERMs could address fibrosis and inflammation together and merits further study.

A limitation of this study is its small sample size: the exploratory CIBERSORTx analyses were based on three donor-matched human skin samples. These data are therefore preliminary and suggestive of E2’s role in IL-6 signaling and fibroblast activation. While our previous publication confirmed E2’s role in IL-6 signaling by defining the positive feed-back loop between IL-6 and E2 [13], future studies would include further verification to establish E2’s capability to influence dermal fibroblast activation and cellular compartments. Another limitation is STAT3 phosphorylation was not assessed, preventing confirmation of direct STAT3 activation. Pharmacologic inhibitors may have off-target effects. Additionally, we did not directly evaluate causality between IL-6 and chemokines in this study. Finally, using genetic approaches to further validate the importance of the IL-6/STAT3 pathway in E2-induced dermal inflammation is desirable.

## 4. Materials and Methods

### 4.1. Human Skin Organ Culture Ex Vivo

Discarded skin tissue was obtained from healthy donors undergoing surgical skin-resection procedures. The demographic information of the donors is summarized in Table 1. Six 3 mm skin punches were placed dermal side down in each well of a 6-well culture plate (Costar, Corning, NY, USA) and maintained in serum-free, phenol-red-free DMEM (Corning, Corning, NY, USA). Tissues were treated with vehicle ethanol (ETOH, Hyclone, South Logan, UT, USA) or E2 (10 nM, Tocris, Minneapolis, MN, USA) for 48 or 72 h. In all experiments, skin tissue and supernatants were harvested at the specified times and stored at −80 °C until further evaluation.

### 4.2. Primary Human Dermal Fibroblast Culture

We established primary human dermal fibroblasts using the outgrowth method [3] from skin tissue isolated from the abdomen, forearm, upper arm, or shoulder of healthy donors or patients with dcSSc under a protocol approved by the IRB of the Medical University of South Carolina. The demographics for healthy donors and dcSSc patients are summarized in Table 1. Dermal fibroblasts were used between passages 3 and 8 and seeded at a density of 1.5–2.0 × 10^5^ cells/well in a 6-well plate (Costar, Corning, NY, USA).

### 4.3. Total RNA Extraction

We extracted total RNA from human skin tissue using the RNeasy^®^ kit (Qiagen, Germantown, MD, USA) according to the manufacturer’s guidelines using chloroform and phenol organic phase extraction. For human skin tissue, one 3 mm skin punch was homogenized in 1 mL of TriZol (Invitrogen, Carlsbad, CA, USA) using an Omni International Bead Ruptor at 4 cycles at 6 m/second for 5 s, with 2 min incubations on ice between cycles.

For primary human dermal fibroblasts, the cells were plated at 2.5 × 10^5^ cells/well in a 6-well culture dish. At the time of harvest, the cells were lysed in 1 mL of TriZol with mechanical disruption (cell scrapping). The total RNA from dermal fibroblasts was further isolated using phenol and chloroform organic phase extraction.

### 4.4. Bulk RNA-Seq of Human Skin Tissue

Total RNA extracted from human skin tissues treated ex vivo with E2 or vehicle ETOH for 48 h was submitted to Novogene Corporation Inc. (Sacramento, CA, USA) for library preparation and sequencing following the workflow previously reported [43]. After quality filtering, paired-end reads were aligned to the Genome Reference Consortium Human Build 38 (GRCh38) using HISAT2 (version 2.0.5) [44].

### 4.5. Gene and Systems Level Analyses

Gene counts were adjusted for batch effects using the ComBat algorithm [45], and DESeq2 was used to perform the differential expression analysis “E2 vs. vehicle” [46]. For each gene, DESeq2 provided the log2 fold change (log2FC) and false discovery rate (FDR)-adjusted *p*-value (q-value). Differentially expressed genes (DEGs) were categorized as upregulated (q < 0.1 and log2FC > 0.6) or downregulated (q < 0.1 and log2FC < −0.6) relative to vehicle ETOH. We generated a heatmap from normalized counts for all transcripts meeting the q-value threshold.

The DEGs were imported into iPathwayGuide (Advaita^®^ Bioinformatics, Ann Arbor, MI, USA), an analytic software that applies the Impact Analysis framework [43]. For the system-level analysis, significance was defined as a *p*-value < 0.05, and all analyses were conducted using default parameters unless otherwise specified.

### 4.6. Transcriptomic Deconvolution of Human Skin RNA-Sequencing Data

Cell-type composition of the bulk RNA-seq samples was estimated using CIBERSORTx digital cytometry [47]. A custom single-cell reference was generated from the publicly available human skin single-cell RNA-seq dataset GSE138669, comprising skin biopsies from 10 healthy controls and 12 patients with SSc [48]. Raw 10x Genomics gene-expression matrices were processed using Scanpy (version 1.12.3). Cells expressing fewer than 600 genes, cells with greater than 25% mitochondrial transcripts, and cells exceeding sample-specific upper thresholds for detected genes or total unique molecular identifier counts and predicted doublets identified using Scrublet were excluded. Following quality control, 40,963 cells from 22 biopsies were retained.

Gene counts were library-size normalized to 10,000 transcripts per cell and log-transformed for clustering and cell-type annotation. Highly variable genes were identified across biopsies, and principal-component analysis, biopsy-level Harmony integration, neighborhood graph construction, Leiden clustering, and uniform manifold approximation and projection were performed. Clusters were assigned to major skin-cell populations based on established marker-gene programs. The resulting reference contained 11 major populations: fibroblasts, keratinocytes, epithelial-appendage cells, blood endothelial cells, lymphatic endothelial cells, pericyte/smooth-muscle cells, melanocytes, myeloid cells, T/NK cells, mast cells, and neural/Schwann cells. Raw single-cell counts, rather than normalized expression values, were supplied to CIBERSORTx. To limit unequal representation while preserving donor diversity, a maximum of 500 cells per major cell population was included in the reference.

A custom signature matrix was generated using the CIBERSORTx single-cell workflow. Quantile normalization was disabled, the minimum expression threshold was set to 0.5, the differential-expression q-value threshold was 0.01, and 300–500 marker genes per cell class were considered. The resulting major-lineage signature matrix contained 4188 genes. Gene-level counts from the six human skin RNA-seq samples were collapsed by HGNC gene symbol, genes with zero counts across all samples were removed, and counts were converted to counts per million. Importantly, the pre-ComBat [45] count matrix was used for deconvolution; ComBat-adjusted and variance-stabilized values were not used.

Relative cell fractions were estimated using CIBERSORTx with S-mode batch correction, quantile normalization disabled, and 100 permutations. CIBERSORTx *p* values, Pearson correlations, and root-mean-square errors were used to evaluate mixture-fit quality. Changes were evaluated within the three donor-matched vehicle and estradiol-treated pairs.

### 4.7. Exploratory Fibroblast-State Deconvolution

To investigate fibroblast heterogeneity, the 10,027 fibroblasts in GSE138669 were further resolved into four transcriptionally distinguishable states based on unsupervised clustering and canonical marker expression (Table S5). These four fibroblast states replaced the single fibroblast category in a refined reference that retained all other major skin-cell populations, resulting in 14 cell classes. To maintain donor representation and reduce reference size, up to 300 cells per class were selected using donor-balanced sampling.

The refined single-cell reference contained 3852 cells and 16,318 expressed genes. CIBERSORTx signature-matrix construction used the same parameters described above and produced a 4500-gene signature matrix with a condition number of 28.06. Relative cell fractions were estimated from the same pre-ComBat CPM-normalized bulk mixture using S-mode batch correction, disabled quantile normalization, and 100 permutations. Fibroblast-state results were considered exploratory because only three donor pairs were available.

### 4.8. Steady-State Transcript Measurement Using qPCR

cDNA was synthesized from 1 µg of RNA following the SuperScript IV First-Strand cDNA Synthesis protocol (Thermofisher Scientific, Catalog #18090050) on a T100 Thermal Cycler (Bio Rad, Hercules, CA, USA). Using undiluted cDNA, we performed qPCR to measure steady-state transcript levels (CFX96 real-time qPCR machine, Bio Rad). Genes of interest, including *CXCL8* (Hs00174103_m1), *CCL20* (Hs00355476_m1), *CXCL2* (Hs00601975_m1), *CXCL3* (Hs0017161_m1) and *IL-6* (Hs00985639_m1), were normalized to *B2M* (Hs00187842_m1). Expression was calculated by the delta-delta CT method and is reported as a fold change over vehicle. All primers were purchased from Thermofisher (Rockford, IL, USA).

### 4.9. CXCL8 ELISA

We harvested the conditioned media of E2- or ETOH-stimulated human skin tissue in organ culture to measure secreted CXCL8, quantified using the human CXCL8 ELISA kit following the manufacturer’s protocol (R and D, Catalog # D8000C), using a Synergy HT Multi-Mode Microplate Reader (Agilent, Winooski, VT, USA).

### 4.10. Immunofluorescence

E2- or vehicle-treated skin tissue in organ culture was harvested after 72 h, preserved in formaldehyde for 24 h, embedded in paraffin, and divided into 6 um sections. The human skin tissue sections were deparaffinized using Histoclear (National Diagnostics, Atlanta, GA, USA) and rehydrated with graded ETOH washes. Heat-induced epitope retrieval was performed in citric acid-based buffer (Vector Laboratories, Newark, CA, USA). Tissue was permeabilized with 1% secondary host serum in PBS + 0.4% Triton X-100 (PBST), blocked with 5% secondary host serum in PBST, and then incubated with CXCL8 (1:200, 27095-1-AP, Proteintech, Rosemont, IL, USA), IL-6 (1:200, Abcam, Waltham, MA, USA), or control IgG antibody (Vector Laboratories, Newark, CA, USA) followed by fluorescently labeled secondary antibodies (1:1500 Alexa Fluor 488 goat anti-rabbit IgG (H + L), A11008, Invitrogen, Eugene, OR, USA). The tissue was then incubated for 10 min at room temperature using a CF^®^ Dye tyramide signaling amplification kit for protein fluorescent labeling. For co-staining, we repeated antigen unmasking and endogenous peroxidase activity blocking steps. Nuclei were counterstained using ProLong Diamond Antifade Mountant with DAPI counterstain (Invitrogen, Eugene, OR, USA). Images were procured with the Invitrogen EVOS M3000 Imaging System, using identical camera settings set based on vehicle treatment and/or isotype control antibody tissue section.

### 4.11. Inhibitor Studies

For STAT3 inhibitor studies, human skin tissue in organ culture was pre-treated with the STAT3 Inhibitor V (Stattic, MilliporeSigma, Burlington, MA, USA) or vehicle DMSO for 1 h prior to E2 (10 nM) or vehicle (ETOH) treatment. For aromatase inhibitor studies, SSc dermal fibroblasts were treated with anastrozole (dissolved in 100% DMSO, Accord, Durham, NC, USA) at 300 nM or an equal volume DMSO vehicle for 48 or 96 h.

### 4.12. Statistical Analysis

All data were tested for normal distribution. Non-normally distributed data were log-transformed to obtain normality. Normally distributed data were analyzed using one-way ANOVA with appropriate post hoc tests or paired or unpaired two-sample *t*-tests, as applicable. For association studies, we used Pearson’s correlation to determine statistical significance, which was defined as a *p*-value < 0.05 using GraphPad (v. 11). Cell-fraction differences were calculated within each donor as the E2-treated fraction minus its matched vehicle fraction and are reported as percentage-point changes. Because only three donor pairs were available, fibroblast-state analyses were interpreted descriptively. Paired *t*-tests and exact two-sided sign-flip tests were calculated for exploratory purposes, without adjustment for multiple comparisons.

## 5. Conclusions

In human skin tissue, E2 induced an inflammatory transcriptional program that included *CXCL2*, *CXCL3*, *CXCL8*, and *CCL20*, and this induction was reduced by STAT3 inhibition. Blocking E2 production only partly reduced chemokine production, which highlights the contribution of endogenous E2 synthesis and points to additional pathways that sustain inflammation. These preliminary findings support further study to establish the causal relationship between IL-6 and chemokine production, confirm STAT3 activation, and determine whether combined targeting of estrogen receptor and STAT3 signaling represents a viable therapeutic approach for SSc-associated skin disease.

## Figures and Tables

**Table 1:** Donor Demographics.

dcSSc Fibroblasts			
**ID**	**Age at Biopsy (Years)**	**Sex**	**Disease Duration (Years)**
SSc 001	54	M	2.5
SSc 002	30	F	4
SSc 003	69	M	3
SSc 005	42	M	5
SSc 006	69	F	2.5
SSc 007	46	M	3
SSc 008	66	F	5
Healthy donor fibroblasts			
ID	Age (years)	Sex	
139	unknown	unknown	
216	34	F	
230	37	F	
333	42	F	
247	24	F	
003	51	M	
Dermal tissue ex vivo			
ID	Age (years)	Sex	
6	47	F	
10	25	F	
225	43	F	
247	24	F	
292	47	F	
303	37	F	
308	47	F	
309	40	F	
313	32	F	
314	46	F	
318	46	F	
319	39	F	
324	44	F	
325	72	F	
331	37	F	
332	54	F	
333	42	F	
401	41	M	
402	45	F	
403	64	M	
404	57	F	
405	53	F	

## Data Availability

The data presented in this study will be deposited in NCBI GEO under accession number GSE338085 and released on 07-08-2027. These data were derived from the following resources available in the NCBI GEO under accession number GSE138669.

## Supplementary Materials

The following supporting information can be downloaded at: https://www.mdpi.com/article/10.3390/ijms27219454/s1.

## Abbreviations

The following abbreviations are used in this manuscript:

SSc	Systemic Sclerosis
E2	Estradiol
RNA-seq	Bulk RNA sequencing
