# Supplementary material for: STAT3 Signaling Contributes to Chemokine Production in E2-Induced Dermal Inflammation
Source: Int J Mol Sci. 2026 Oct 23;27(21):9454. doi: 10.3390/ijms27219454 (PMC13763398; doi:10.3390/ijms27219454)
Supplement: Supplementary file 1 [file ijms-27-09454-s001.zip › ijms-4567736-supplementary.pdf]

Supplemental Figure S1

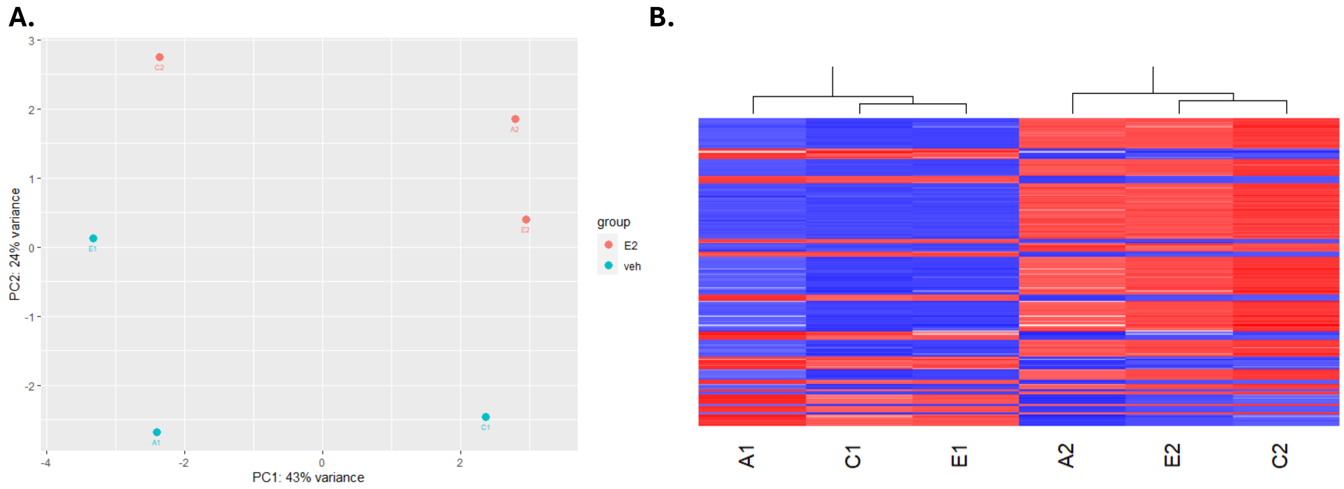

PCA and heat map of ETOH- or E2-treated human skin tissue. **(A) PCA plot:** Clustering of the human skin tissue samples by treatment post batch effect correction using the ComBat algorithm. **(B) Volcano plot:** All 90 significantly differentially expressed (DE) genes are represented in terms of their measured expression change (x-axis) and the significance of the change (y-axis). The significance is represented in terms of the negative log (base 10) of the p-value, so that more significant genes are plotted higher on the y-axis. The up-regulated genes (positive log fold change) are shown in red, while the down-regulated genes are blue.

Supplementary Table S1*: 90 Differentially Expressed Genes*

Symbol	Entrez	logfc	adjpv
**APLNR**	187	1.09002431	0.000001
**COL4A1**	1282	0.71293944	0.000001
**COL4A2**	1284	0.74057444	0.000001
**CSF3**	1440	1.29722074	0.000001
**CTSK**	1513	-0.6460228	0.000001
**GJA4**	2701	1.17128145	0.000001
**CXCL2**	2920	1.22628853	0.000001
**CXCL3**	2921	1.3304411	0.000001
**HLX**	3142	0.64493109	0.000001
**HSPG2**	3339	0.75124113	0.000001
**CXCL8**	3576	1.04360745	0.000001
**KRT1**	3848	-0.6107985	0.000001
**LIF**	3976	1.06042171	0.000001
**LOXL2**	4017	0.71622703	0.000001
**PDGFB**	5155	0.87643308	0.000001
**CCL20**	6364	2.06264699	0.000001
**CXCL6**	6372	1.52580049	0.000001
**CXCL5**	6374	2.08890061	0.000001
**VWF**	7450	0.78460172	0.000001
**WNT5A**	7474	1.01467518	0.000001
**CXCR4**	7852	0.63357702	0.000001
**TFPI2**	7980	0.78381938	0.000001
**CHST1**	8534	1.14059368	0.000001
**TP53I11**	9537	0.63579236	0.000001
**CDHR1**	92211	-0.6214566	0.000001
**TNFAIP8L1**	126282	0.87935954	0.000001
**RPL7AP6**	326310	-0.6353585	0.000001
**C11orf96**	387763	0.8220654	0.000001
**C2CD4B**	388125	0.6263864	0.000001
**NPIPB4**	440345	-2.6101174	0.000001
**NR4A1**	3164	0.64348678	0.00000196
**BCL9L**	283149	0.76377124	0.00000197
**MRGPRF**	116535	0.79813206	0.000004
**SYDE1**	85360	0.62508809	0.00000691
**PDK4**	5166	1.16678647	0.00000857
**FLT4**	2324	0.84360268	0.00000913

**CXorf36**	79742	0.75940654	0.0000178
**PEAR1**	375033	0.968192	0.0000524
**MYOM3**	127294	-0.7609146	0.0000706
**GPIHBP1**	338328	0.98432742	0.0000709
**IL1B**	3553	1.06181043	0.00010496
**EXOC3L2**	90332	1.01577042	0.00010597
**EPOR**	2057	0.72972651	0.00013613
**RRAD**	6236	0.95964243	0.00026672
**FGF2**	2247	0.80822851	0.00028709
**SOX17**	64321	0.79713186	0.00028709
**H19**	283120	1.11819481	0.00028943
**CDH11**	1009	0.73428066	0.00031229
**AOC1**	26	0.66755676	0.00032024
**TBXA2R**	6915	1.22677818	0.00042185
**HID1**	283987	0.64594386	0.00053175
**IL11**	3589	0.7314205	0.00053329
**CD300E**	342510	-1.4170125	0.00060564
**CBWD4P**	653510	-1.5667716	0.00067614
**PCDHGC3**	5098	1.06831412	0.00071674
**RAVER1**	125950	0.7689898	0.00076442
**CKAP2**	26586	-0.6342067	0.0008811
**CHST2**	9435	0.72837348	0.0011031
**CCDC188**	388849	1.76991335	0.0011031
**ADGRA2**	25960	0.71224277	0.00128839
**CLEC14A**	161198	0.68171263	0.00133295
**OLFML2B**	25903	0.68506968	0.00189554
**RASSF4**	83937	0.71206221	0.00215655
**WNT11**	7481	1.06108503	0.00260133
**KLHL5**	51088	0.62485819	0.003764
**BAALC**	79870	1.47653305	0.003764
**CYTL1**	54360	0.71699797	0.00410053
**HSPA7**	3311	0.66961382	0.00414075
**GIMAP8**	155038	0.79142812	0.00414075
**NPR1**	4881	0.65255492	0.0057007
**DCD**	117159	-1.5736572	0.00634086
**MALL**	7851	0.68352632	0.006651
**EIF3C**	8663	0.843207	0.00719115
**SLC29A4**	222962	0.75112587	0.00778639
**TM4SF18**	116441	0.73735461	0.0093288

**LTB**	4050	-0.6367643	0.01378227
**ABI3BP**	25890	0.61722679	0.019063
**GPR173**	54328	1.27158479	0.0215394
**ADAM12**	8038	0.78160943	0.02594356
**ARC**	23237	1.07383382	0.02594356
**SLC9A3**	6550	1.26048992	0.02848018
**CCL5**	6352	-0.9778648	0.02922466
**FBXL14**	144699	0.95413348	0.02995648
**MEF2C**	4208	0.74004249	0.03021878
**GNPNAT1**	64841	0.60926669	0.03036903
**ITGA9**	3680	0.79408265	0.03152881
**PFN2**	5217	0.64840398	0.03344266
**ESM1**	11082	0.6352525	0.04143765
**MAOB**	4129	-0.8600618	0.04879014
**RGL3**	57139	0.7258587	0.04936274

***Supplemental Table S2**: Impacted Pathways*

Pathway Name	count DE	count All	p-value	Pathway Accumulation p-value	pComb	Over-Representation Analysis p-value
**Cytokine-cytokine receptor interaction**	14	141	1.15857867924479e-14	0.00049975	1.15857867924479e-14	6.31839303428745e-13
**Rheumatoid arthritis**	11	67	4.79329030446033e-12	0.20089955	4.79329030446033e-12	7.82756925917929e-13
**Viral protein interaction with cytokine and cytokine receptor**	8	49	1.84439713795122e-11	0.00049975	1.84439713795122e-11	1.26884674705416e-9
**IL-17 signaling pathway**	8	73	1.42928822968451e-8	0.019490255	1.42928822968451e-8	3.30899220284588e-8
**Chemokine signaling pathway**	8	130	3.10604681350994e-8	0.00049975	3.10604681350994e-8	2.91133E-06
**TNF signaling pathway**	8	103	7.31467087422977e-7	0.08195902	7.31467087422977e-7	4.95280243235685e-7
**PI3K-Akt signaling pathway**	10	250	6.92706E-05	0.671664168	6.92706E-05	7.84027E-06
**Amoebiasis**	6	76	0.000141792	0.88055972	0.000141792	1.301E-05
**JAK-STAT signaling pathway**	5	101	0.00030524	0.04147926	0.00030524	0.000637669
**Calcium signaling pathway**	5	138	0.000309675	0.010494753	0.000309675	0.002560431
**ECM-receptor interaction**	5	60	0.000372373	0.6001999	0.000372373	5.47954E-05
**Pathways in cancer**	10	411	0.00044297	0.08045977	0.00044297	0.000494572
**Legionellosis**	4	48	0.000499102	0.142428786	0.000499102	0.000318547
**Toll-like receptor signaling pathway**	4	70	0.000735604	0.051974013	0.000735604	0.001338609
**NOD-like receptor signaling pathway**	5	139	0.001287737	0.048975512	0.001287737	0.00264184
**NF-kappa B signaling pathway**	5	83	0.001535175	0.611194403	0.001535175	0.000257429

**Malaria**	3	27	0.001620598	0.206896552	0.001620598	0.000807785
**AGE-RAGE signaling pathway in diabetic complications**	4	84	0.001715155	0.067966017	0.001715155	0.002619555
**Pertussis**	4	58	0.002081951	0.335332334	0.002081951	0.000659302
**Lipid and atherosclerosis**	5	166	0.002090288	0.03948026	0.002090288	0.005624986
**Hematopoietic cell lineage**	4	54	0.002330935			0.00050179
**Kaposi sarcoma-associated herpesvirus infection**	5	156	0.002880403	0.073463268	0.002880403	0.004331066
**Alcoholic liver disease**	4	104	0.003259756	0.064967516	0.003259756	0.005629
**Focal adhesion**	6	162	0.004533626	0.624187906	0.004533626	0.000850384
**Epithelial cell signaling in Helicobacter pylori infection**	4	61	0.00459267	0.674662669	0.00459267	0.000798379
**Chagas disease**	3	75	0.005619369	0.045977011	0.005619369	0.014729912
**Breast cancer**	4	117	0.006090928	0.087456272	0.006090928	0.008487345
**Proteoglycans in cancer**	4	171	0.007178614	0.029985007	0.007178614	0.029857226
**MAPK signaling pathway**	6	233	0.008489452	0.205897051	0.008489452	0.005268238
**Gastric cancer**	3	111	0.008708641	0.027486257	0.008708641	0.040634585
**Regulation of actin cytoskeleton**	5	170	0.009861301	0.207396302	0.009861301	0.006211849
**Glycosaminoglycan biosynthesis - keratan sulfate**	2	12	0.011027253			0.002930554
**Cytoskeleton in muscle cells**	5	155	0.015093286			0.004214623

**Basal cell carcinoma**	2	55	0.015380354	0.03948026	0.015380354	0.054558378
**Coronavirus disease - COVID-19**	4	174	0.016089226	0.071964018	0.016089226	0.03154245
**Cushing syndrome**	3	118	0.016651625	0.049975012	0.016651625	0.047275751
**Amphetamine addiction**	2	44	0.020149927	0.08095952	0.020149927	0.036466321
**Melanoma**	2	52	0.020338283	0.060469765	0.020338283	0.049357905
**Protein digestion and absorption**	3	54	0.020417			0.00599641
**Histidine metabolism**	2	18	0.022197419			0.006614624
**Signaling pathways regulating pluripotency of stem cells**	4	111	0.023494809			0.007071625
**Melanogenesis**	2	82	0.024055707	0.033483258	0.024055707	0.108574372
**Alzheimer disease**	3	310	0.024699429	0.010494753	0.024699429	0.357354372
**Human papillomavirus infection**	6	272	0.025141503	0.351324338	0.025141503	0.01090057
**Hippo signaling pathway**	2	135	0.029398749	0.019490255	0.029398749	0.236424774
**mTOR signaling pathway**	2	139	0.034350856	0.022488756	0.034350856	0.246570787
**Transcriptional misregulation in cancer**	2	137	0.0362537	0.024487756	0.0362537	0.241495077
**Type I diabetes mellitus**	1	24	0.0371381	0.03998001	0.0371381	0.152239619
**Graft-versus-host disease**	1	22	0.039026613	0.045977011	0.039026613	0.140481187
**Mineral absorption**	1	38	0.040101578	0.028985507	0.040101578	0.230211235
**Hepatocellular carcinoma**	2	145	0.040757176	0.025987006	0.040757176	0.2618197

**Tryptophan metabolism**	2	30	0.051001312			0.017833108
**Influenza A**	3	131	0.05188509	0.149425287	0.05188509	0.060928217
**Phospholipase D signaling pathway**	2	113	0.055491246	0.054472764	0.055491246	0.181346766
**Arrhythmogenic right ventricular cardiomyopathy**	1	52	0.056425633	0.033483258	0.056425633	0.301083887
**Rap1 signaling pathway**	4	161	0.056489815	0.409795102	0.056489815	0.024634831
**Cytosolic DNA-sensing pathway**	2	59	0.056774784	0.164417791	0.056774784	0.061777271
**Virion - Human immunodeficiency virus**	1	3	0.057009231			0.020416786
**Yersinia infection**	2	116	0.070757039	0.070464768	0.070757039	0.188750603
**Osteoclast differentiation**	2	103	0.071947961	0.086456772	0.071947961	0.157033456
**MicroRNAs in cancer**	1	147	0.072530354	0.021489255	0.072530354	0.638096641
**Arginine and proline metabolism**	2	39	0.076304917			0.029197447
**Choline metabolism in cancer**	1	81	0.080563329	0.036481759	0.080563329	0.42800481
**Pathways of neurodegeneration - multiple diseases**	3	370	0.082673045	0.034482759	0.082673045	0.46758264
**Relaxin signaling pathway**	2	96	0.082745785	0.114942529	0.082745785	0.140428136
**Gap junction**	1	60	0.084572223	0.048975512	0.084572223	0.338643862
**Dilated cardiomyopathy**	1	59	0.087062699	0.051474263	0.087062699	0.334060306
**Bile secretion**	1	33	0.091029465	0.089455272	0.091029465	0.203214239

**Intestinal immune network for IgA production**	1	24	0.100353799	0.134932534	0.100353799	0.152239619
**African trypanosomiasis**	1	18	0.105758466	0.188405797	0.105758466	0.116478381
**Oxytocin signaling pathway**	2	107	0.108152684	0.135432284	0.108152684	0.166684264
**Human cytomegalovirus infection**	4	177	0.110089044	0.693653173	0.110089044	0.033282828
**Prostate cancer**	1	85	0.113301778	0.053973013	0.113301778	0.443618544
**cGMP-PKG signaling pathway**	2	109	0.115443629	0.142928536	0.115443629	0.171548496
**EGFR tyrosine kinase inhibitor resistance**	2	72	0.116990103	0.285857071	0.116990103	0.087236468
**Fluid shear stress and atherosclerosis**	3	117	0.121079539	0.562718641	0.121079539	0.046296029
**Cocaine addiction**	1	32	0.12227608	0.133433283	0.12227608	0.197703541
**Th17 cell differentiation**	1	81	0.13047464	0.066966517	0.13047464	0.42800481
**Neuroactive ligand-receptor interaction**	2	93	0.132173817	0.218390805	0.132173817	0.133437216
**Antifolate resistance**	1	25	0.14047853	0.1994003	0.14047853	0.15805904
**Wnt signaling pathway**	3	139	0.142045919	0.455772114	0.142045919	0.070147619
**Phenylalanine metabolism**	1	10	0.146366444			0.066466746
**Platelet activation**	2	97	0.150770752	0.24187906	0.150770752	0.142776087
**Aldosterone synthesis and secretion**	2	63	0.150986647	0.499250375	0.150986647	0.069301315

**Proximal tubule bicarbonate reclamation**	1	12	0.167507574			0.079226077
**Small cell lung cancer**	2	84	0.169055964	0.354822589	0.169055964	0.112995972
**Regulation of lipolysis in adipocytes**	1	36	0.174154882	0.189905047	0.174154882	0.219522738
**Hepatitis B**	2	135	0.180551146	0.184907546	0.180551146	0.236424774
**Non-alcoholic fatty liver disease**	2	134	0.183828915	0.191404298	0.183828915	0.233891995
**Bladder cancer**	1	38	0.188052523	0.2003998	0.188052523	0.230211235
**Salmonella infection**	3	216	0.191425014	0.255872064	0.191425014	0.184603337
**Glioma**	1	63	0.192843784	0.135432284	0.192843784	0.352208169
**Renin secretion**	1	41	0.196181045	0.1984008	0.196181045	0.245973137
**Shigellosis**	4	206	0.198855953	0.938030985	0.198855953	0.052972301
**C-type lectin receptor signaling pathway**	1	86	0.200056115	0.111944028	0.200056115	0.447455658
**Serotonergic synapse**	1	57	0.207482853	0.16191904	0.207482853	0.324798749
**Diabetic cardiomyopathy**	1	163	0.207859473	0.077961019	0.207859473	0.67622294
**Inflammatory bowel disease**	1	42	0.208220865	0.210394803	0.208220865	0.251155848
**RIG-I-like receptor signaling pathway**	1	52	0.216035391	0.184407796	0.216035391	0.301083887
**Complement and coagulation cascades**	1	43	0.219183135	0.220889555	0.219183135	0.256303329
**Leishmaniasis**	1	58	0.22207265	0.174912544	0.22207265	0.32944534
**Tyrosine metabolism**	1	21	0.248836692			0.134541638

**Glycine, serine and threonine metabolism**	1	22	0.256787591			0.140481187
**Dopaminergic synapse**	1	88	0.25747006	0.154922539	0.25747006	0.455051549
**Prion disease**	2	212	0.276595541	0.181909045	0.276595541	0.427954062
**Cellular senescence**	1	139	0.277140362	0.126436782	0.277140362	0.617401312
**Renal cell carcinoma**	1	63	0.288462525	0.234382809	0.288462525	0.352208169
**Alcoholism**	1	90	0.29183939	0.181409295	0.29183939	0.462544162
**Leukocyte transendothelial migration**	1	84	0.309605242	0.207396302	0.309605242	0.439755082
**Vascular smooth muscle contraction**	1	87	0.311000144	0.203398301	0.311000144	0.4512666
**Drug metabolism - cytochrome P450**	1	30	0.314564583			0.18656883
**Neutrophil extracellular trap formation**	1	92	0.319439563	0.202898551	0.319439563	0.469934888
**Measles**	1	104	0.322745686	0.188905547	0.322745686	0.512215049
**Ras signaling pathway**	3	166	0.327175308	0.935532234	0.327175308	0.105463892
**Amyotrophic lateral sclerosis**	1	289	0.328174185	0.114442779	0.328174185	0.865904939
**Biosynthesis of nucleotide sugars**	1	33	0.333946194			0.203214239
**Hippo signaling pathway - multiple species**	1	26	0.334890969	0.622688656	0.334890969	0.163838955
**Thermogenesis**	1	186	0.33826704	0.142928536	0.33826704	0.724169054
**Tuberculosis**	1	131	0.345791418	0.179410295	0.345791418	0.595536416
**Necroptosis**	1	106	0.35726862	0.215892054	0.35726862	0.518929916
**Parkinson disease**	1	216	0.366715136	0.149925037	0.366715136	0.776298093

**Pathogenic Escherichia coli infection**	2	161	0.389643612	0.420789605	0.389643612	0.302470839
**Amino sugar and nucleotide sugar metabolism**	1	44	0.396671074			0.261415818
**Chemical carcinogenesis - receptor activation**	1	129	0.417621021	0.23938031	0.417621021	0.589879871
**Apelin signaling pathway**	2	99	0.419400474	0.963518241	0.419400474	0.147497103
**Herpes simplex virus 1 infection**	2	398	0.441096788	0.2013993	0.441096788	0.761980426
**Viral life cycle - HIV-1**	1	56	0.453323271			0.32012032
**Hypertrophic cardiomyopathy**	1	57	0.457592182			0.324798749
**Inflammatory mediator regulation of TRP channels**	1	71	0.511359939			0.387049307
**Axon guidance**	2	142	0.537712317	0.826086957	0.537712317	0.254192136
**Cell adhesion molecules**	1	87	0.561566122			0.4512666
**Cortisol synthesis and secretion**	1	41	0.571973767	0.946026987	0.571973767	0.245973137
**Purine metabolism**	1	93	0.577914301			0.473592471
**Lysosome**	1	122	0.642411128			0.569458949
**Parathyroid hormone synthesis, secretion and action**	1	87	0.657851375	0.658670665	0.657851375	0.4512666
**Endocytosis**	1	226	0.762556646			0.791407765
**cAMP signaling pathway**	1	124	0.76385865	0.690154923	0.76385865	0.575393965

**Metabolic pathways**	4	1137	0.831728813			0.958431133
**Human immunodeficiency virus 1 infection**	1	166	0.855158856	0.750624688	0.855158856	0.68291592
**Apoptosis**	1	119	0.871676357	0.96051974	0.871676357	0.560402283
**Efferocytosis**	1	129	0.893925144	0.977011494	0.893925144	0.589879871

DE: Differential Expression, pComb: Combines pORA and pAcc

Supplemental Table S3: *Gene Ontology (GO) terms*

goId	goName	count DE	count All	pv	pv_fdr	pv_Bonferroni	pv_high_ specifcity _ pruning	pv_smallest_common_denominator_ pruning
**GO:0005125**	cytokine activity	15	102	1.1e-16	3.718e-14	3.718e-14	5.577E-06	3.718e-14
**GO:0030545**	signaling receptor regulator activity	17	218	4.4e-14	7.436e-12	1.4872e-11	1	1
**GO:0048018**	receptor ligand activity	16	200	1.8e-13	2.028e-11	6.084e-11	1	1
**GO:0030546**	signaling receptor activator activity	16	204	2.4e-13	2.028e-11	8.112e-11	1	1
**GO:0008009**	chemokine activity	7	19	2e-11	1.352e-9	6.76e-9	6.76e-9	1
**GO:0005126**	cytokine receptor binding	12	153	3.1e-10	1.74633333333333e -8	1.0478e-7	0.48780532	0.07715724
**GO:0042379**	chemokine receptor binding	7	30	7.6e-10	3.66971428571429e -8	2.5688e-7	1	1
**GO:0045236**	CXCR chemokine receptor binding	5	10	2.7e-9	1.14075e-7	9.126e-7	6.5347E-06	4.563e-7
**GO:0140677**	molecular function activator activity	19	695	8.7e-8	3.2673E-06	2.9406E-05	1	1
**GO:0005102**	signaling receptor binding	21	873	1.4e-7	4.732E-06	0.00004732	1	1
**GO:0001664**	G protein-coupled receptor binding	9	152	6.7e-7	2.0587E-05	0.00022646	1	1
**GO:0042056**	chemoattractant activity	4	20	0.000007 9	0.00022252	0.0026702	0.01352	0.00089007
**GO:0098772**	molecular function regulator activity	23	1361	0.000015	0.00039	0.00507	1	1
**GO:0038023**	signaling receptor activity	13	512	0.000028	0.00063093	0.009464	0.30569795	0.59685532
**GO:0060089**	molecular transducer activity	13	512	0.000028	0.00063093	0.009464	0.30569795	1
**GO:0070851**	growth factor receptor binding	6	94	0.000035	0.00073938	0.01183	0.14181617	0.0029575
**GO:0004930**	G protein-coupled receptor activity	7	142	0.000041	0.00081518	0.013858	0.26496459	0.297375
**GO:0016641**	oxidoreductase activity, acting on the CH-NH2 group of	3	13	0.000076	0.00142711	0.025688	1	1

	donors, oxygen as acceptor							
**GO:0005201**	extracellular matrix structural constituent	6	111	0.00009	0.00160105	0.03042	0.07060444	0.00563333
**GO:0016638**	oxidoreductase activity, acting on the CH-NH2 group of donors	3	14	0.000096	0.00160952	0.032448	1	1
**GO:0008083**	growth factor activity	5	71	0.0001	0.00160952	0.0338	0.00845	0.00563333
**GO:0004888**	transmembrane signaling receptor activity	10	377	0.00018	0.00276545	0.06084	0.55503788	1
**GO:0001517**	N-acetylglucosamine 6-O-sulfotransferase activity	2	4	0.00026	0.00382087	0.08788	0.01464667	0.01255429
**GO:0005518**	collagen binding	4	59	0.00061	0.00859083	0.20618	0.07458533	0.02403556
**GO:0008131**	primary amine oxidase activity	2	6	0.00064	0.0086528	0.21632	0.14181617	0.02403556
**GO:0005178**	integrin binding	5	111	0.00083	0.01079	0.28054	0.04007714	0.028054
**GO:0048407**	platelet-derived growth factor binding	2	10	0.00188	0.02353481	0.63544	0.07060444	0.05776727
**GO:0060229**	lipase activator activity	2	12	0.00273	0.032955	0.92274	0.28010259	0.076895
**GO:0030547**	signaling receptor inhibitor activity	2	14	0.00373	0.04303	1	1	1
**GO:0048020**	CCR chemokine receptor binding	2	15	0.00429	0.04303	1	1	0.07715724
**GO:0019956**	chemokine binding	2	16	0.00488	0.04303	1	0.07458533	0.07715724
**GO:0008201**	heparin binding	4	104	0.00495	0.04303	1	0.07458533	0.07715724
**GO:0004343**	glucosamine 6-phosphate N-acetyltransferase activity	1	1	0.00662	0.04303	1	0.07458533	0.07715724
**GO:0004900**	erythropoietin receptor activity	1	1	0.00662	0.04303	1	0.07458533	0.07715724
**GO:0004960**	thromboxane receptor activity	1	1	0.00662	0.04303	1	1	1
**GO:0004961**	thromboxane A2 receptor activity	1	1	0.00662	0.04303	1	0.07458533	0.07715724

**GO:0004968**	gonadotropin-releasing hormone receptor activity	1	1	0.00662	0.04303	1	0.07458533	0.07715724
**GO:0005115**	receptor tyrosine kinase-like orphan receptor binding	1	1	0.00662	0.04303	1	0.07458533	0.07715724
**GO:0005130**	granulocyte colony-stimulating factor receptor binding	1	1	0.00662	0.04303	1	0.07458533	1
**GO:0005142**	interleukin-11 receptor binding	1	1	0.00662	0.04303	1	0.07458533	1
**GO:0031726**	CCR1 chemokine receptor binding	1	1	0.00662	0.04303	1	0.07458533	1
**GO:0031729**	CCR4 chemokine receptor binding	1	1	0.00662	0.04303	1	0.07458533	1
**GO:0035478**	chylomicron binding	1	1	0.00662	0.04303	1	0.07458533	0.07715724
**GO:0038147**	C-X-C motif chemokine 12 receptor activity	1	1	0.00662	0.04303	1	0.07458533	0.07715724
**GO:0045130**	keratan sulfotransferase activity	1	1	0.00662	0.04303	1	0.07458533	0.07715724
**GO:0046817**	chemokine receptor antagonist activity	1	1	0.00662	0.04303	1	0.07458533	0.07715724
**GO:0052597**	diamine oxidase activity	1	1	0.00662	0.04303	1	1	1
**GO:0052598**	histamine oxidase activity	1	1	0.00662	0.04303	1	0.07458533	0.07715724
**GO:0052599**	methylputrescine oxidase activity	1	1	0.00662	0.04303	1	0.07458533	0.07715724
**GO:0052600**	propane-1,3-diamine oxidase activity	1	1	0.00662	0.04303	1	0.07458533	0.07715724
**GO:0060182**	apelin receptor activity	1	1	0.00662	0.04303	1	0.07458533	0.07715724
**GO:0060230**	lipoprotein lipase activator activity	1	1	0.00662	0.04303	1	0.07458533	1
**GO:0008146**	sulfotransferase activity	2	23	0.00999	0.06370981	1	1	1
**GO:0005146**	leukemia inhibitory factor receptor binding	1	2	0.01319	0.07686586	1	0.12737771	1
**GO:0005171**	hepatocyte growth factor receptor binding	1	2	0.01319	0.07686586	1	0.12737771	1

**GO:0030550**	acetylcholine receptor inhibitor activity	1	2	0.01319	0.07686586	1	0.12737771	0.14381355
**GO:0090722**	receptor-receptor interaction	1	2	0.01319	0.07686586	1	0.12737771	0.14381355
**GO:1902379**	chemoattractant activity involved in axon guidance	1	2	0.01319	0.07686586	1	0.12737771	1
**GO:0005539**	glycosaminoglycan binding	4	143	0.01482	0.08490102	1	1	1
**GO:0030246**	carbohydrate binding	4	144	0.01517	0.08545767	1	0.27681772	0.16023313
**GO:0005109**	frizzled binding	2	31	0.01775	0.09387831	1	0.14181617	0.18180303
**GO:0030020**	extracellular matrix structural constituent conferring tensile strength	2	31	0.01775	0.09387831	1	0.14181617	1
**GO:0005198**	structural molecule activity	8	507	0.01859	0.09387831	1	1	1
**GO:0005153**	interleukin-8 receptor binding	1	3	0.01972	0.09387831	1	0.14181617	1
**GO:0016941**	natriuretic peptide receptor activity	1	3	0.01972	0.09387831	1	0.14181617	0.18514889
**GO:0030298**	receptor signaling protein tyrosine kinase activator activity	1	3	0.01972	0.09387831	1	0.14181617	0.18514889
**GO:0031731**	CCR6 chemokine receptor binding	1	3	0.01972	0.09387831	1	0.14181617	1
**GO:0052596**	phenethylamine:oxygen oxidoreductase (deaminating) activity	1	3	0.01972	0.09387831	1	0.14181617	1
**GO:0070052**	collagen V binding	1	3	0.01972	0.09387831	1	0.14181617	1
**GO:0070492**	oligosaccharide binding	1	3	0.01972	0.09387831	1	0.14181617	1
**GO:0097621**	monoamine oxidase activity	1	3	0.01972	0.09387831	1	0.14181617	0.18514889
**GO:0008528**	G protein-coupled peptide receptor activity	2	35	0.02233	0.10482694	1	0.58112889	1
**GO:0008504**	monoamine transmembrane transporter activity	1	4	0.0262	0.11652105	1	0.177112	0.22706667

**GO:0016494**	C-X-C chemokine receptor activity	1	4	0.0262	0.11652105	1	1	1
**GO:0035473**	lipase binding	1	4	0.0262	0.11652105	1	0.177112	0.22706667
**GO:0052595**	aliphatic amine oxidase activity	1	4	0.0262	0.11652105	1	0.177112	0.22706667
**GO:0016782**	transferase activity, transferring sulphur-containing groups	2	39	0.02734	0.12001195	1	1	1
**GO:1901681**	sulfur compound binding	4	175	0.02862	0.12262127	1	1	1
**GO:0001653**	peptide receptor activity	2	40	0.02866	0.12262127	1	1	1
**GO:0004383**	guanylate cyclase activity	1	5	0.03265	0.12832209	1	0.19360877	0.24523778
**GO:0004720**	protein-lysine 6-oxidase activity	1	5	0.03265	0.12832209	1	0.19360877	0.24523778
**GO:0004740**	pyruvate dehydrogenase (acetyl-transferring) kinase activity	1	5	0.03265	0.12832209	1	0.19360877	0.24523778
**GO:0019534**	toxin transmembrane transporter activity	1	5	0.03265	0.12832209	1	0.19360877	0.24523778
**GO:0031730**	CCR5 chemokine receptor binding	1	5	0.03265	0.12832209	1	0.19360877	1
**GO:0032027**	myosin light chain binding	1	5	0.03265	0.12832209	1	0.19360877	0.24523778
**GO:1990430**	extracellular matrix protein binding	1	5	0.03265	0.12832209	1	0.19360877	0.24523778
**GO:0050840**	extracellular matrix binding	2	45	0.03561	0.1383469	1	0.20752034	0.26165609
**GO:0019838**	growth factor binding	3	112	0.03799	0.14591614	1	0.88294495	1
**GO:0005021**	vascular endothelial growth factor receptor activity	1	6	0.03905	0.14665444	1	0.21998167	0.27497708
**GO:0005337**	nucleoside transmembrane transporter activity	1	6	0.03905	0.14665444	1	0.21998167	0.27497708
**GO:0005507**	copper ion binding	2	48	0.04007	0.14883143	1	0.22202721	0.27640122

**GO:0003680**	minor groove of adenine-thymine-rich DNA binding	1	7	0.04541	0.15988104	1	0.24362825	0.297375
**GO:0004954**	prostanoid receptor activity	1	7	0.04541	0.15988104	1	1	1
**GO:0016176**	superoxide-generating NADPH oxidase activator activity	1	7	0.04541	0.15988104	1	0.24362825	0.297375
**GO:0030548**	acetylcholine receptor regulator activity	1	7	0.04541	0.15988104	1	1	1
**GO:0099602**	neurotransmitter receptor regulator activity	1	7	0.04541	0.15988104	1	1	1
**GO:0046982**	protein heterodimerization activity	4	206	0.04749	0.16548062	1	0.25080656	0.30286075
**GO:0004896**	cytokine receptor activity	2	55	0.05122	0.16975476	1	1	1
**GO:0005509**	calcium ion binding	6	405	0.05132	0.16975476	1	0.25340203	0.30674982
**GO:0005104**	fibroblast growth factor receptor binding	1	8	0.05173	0.16975476	1	0.25340203	1
**GO:0005326**	neurotransmitter transmembrane transporter activity	1	8	0.05173	0.16975476	1	0.25340203	0.30674982
**GO:0016493**	C-C chemokine receptor activity	1	8	0.05173	0.16975476	1	0.25340203	0.30674982
**GO:0019865**	immunoglobulin binding	1	8	0.05173	0.16975476	1	0.25340203	0.30674982
**GO:0001637**	G protein-coupled chemoattractant receptor activity	1	9	0.05801	0.17664306	1	1	1
**GO:0004950**	chemokine receptor activity	1	9	0.05801	0.17664306	1	1	1
**GO:0005243**	gap junction channel activity	1	9	0.05801	0.17664306	1	0.26496459	0.32678967
**GO:0015386**	potassium:proton antiporter activity	1	9	0.05801	0.17664306	1	0.26496459	0.32678967
**GO:0016500**	protein-hormone receptor activity	1	9	0.05801	0.17664306	1	1	1
**GO:0019911**	structural constituent of myelin sheath	1	9	0.05801	0.17664306	1	0.26496459	0.32678967

**GO:0019957**	C-C chemokine binding	1	9	0.05801	0.17664306	1	0.26496459	1
**GO:0048019**	receptor antagonist activity	1	9	0.05801	0.17664306	1	1	1
**GO:0004016**	adenylate cyclase activity	1	10	0.06424	0.18880974	1	0.27681772	0.3446527
**GO:0015385**	sodium:proton antiporter activity	1	10	0.06424	0.18880974	1	0.27681772	0.3446527
**GO:0015562**	efflux transmembrane transporter activity	1	10	0.06424	0.18880974	1	0.27681772	0.3446527
**GO:0016004**	phospholipase activator activity	1	10	0.06424	0.18880974	1	0.27681772	1
**GO:0042802**	identical protein binding	15	1516	0.07025	0.19676628	1	0.28010259	0.366288
**GO:0004953**	icosanoid receptor activity	1	11	0.07044	0.19676628	1	1	1
**GO:0005161**	platelet-derived growth factor receptor binding	1	11	0.07044	0.19676628	1	0.28010259	1
**GO:0022821**	solute:potassium antiporter activity	1	11	0.07044	0.19676628	1	0.28010259	1
**GO:0030021**	extracellular matrix structural constituent conferring compression resistance	1	11	0.07044	0.19676628	1	0.28010259	1
**GO:0035259**	nuclear glucocorticoid receptor binding	1	11	0.07044	0.19676628	1	0.28010259	0.366288
**GO:0050839**	cell adhesion molecule binding	6	444	0.07328	0.20302164	1	1	1
**GO:0008013**	beta-catenin binding	2	68	0.07441	0.20447626	1	0.2924486	0.38106939
**GO:0140375**	immune receptor activity	2	69	0.07631	0.20800629	1	1	1
**GO:0005149**	interleukin-1 receptor binding	1	13	0.08271	0.22364784	1	0.31411213	1
**GO:0008047**	enzyme activator activity	6	467	0.08842	0.23443469	1	0.9467042	1
**GO:0009975**	cyclase activity	1	14	0.08878	0.23443469	1	1	1
**GO:0016849**	phosphorus-oxygen lyase activity	1	14	0.08878	0.23443469	1	1	1
**GO:0048038**	quinone binding	1	15	0.09482	0.2484431	1	0.35610178	0.47834567

**GO:0004435**	phosphatidylinositol phospholipase C activity	1	16	0.10081	0.26010519	1	0.37036717	0.501085
**GO:0051139**	metal cation:proton antiporter activity	1	16	0.10081	0.26010519	1	0.37036717	1
**GO:0030280**	structural constituent of skin epidermis	1	17	0.10677	0.27339591	1	0.38804581	0.52301826
**GO:0001530**	lipopolysaccharide binding	1	19	0.11856	0.29173319	1	0.40891102	0.54894904
**GO:0003785**	actin monomer binding	1	19	0.11856	0.29173319	1	0.40891102	0.54894904
**GO:0004629**	phospholipase C activity	1	19	0.11856	0.29173319	1	0.40891102	1
**GO:0008656**	cysteine-type endopeptidase activator activity involved in apoptotic process	1	19	0.11856	0.29173319	1	0.40891102	0.54894904
**GO:0050750**	low-density lipoprotein particle receptor binding	1	19	0.11856	0.29173319	1	0.40891102	0.54894904
**GO:0046983**	protein dimerization activity	8	746	0.11911	0.29173319	1	0.88294495	1
**GO:0022829**	wide pore channel activity	1	20	0.12441	0.30036129	1	0.4205058	1
**GO:0070325**	lipoprotein particle receptor binding	1	20	0.12441	0.30036129	1	0.4205058	1
**GO:0017046**	peptide hormone binding	1	21	0.13021	0.30563181	1	0.43575228	0.59474297
**GO:0030296**	protein tyrosine kinase activator activity	1	21	0.13021	0.30563181	1	1	1
**GO:0071813**	lipoprotein particle binding	1	21	0.13021	0.30563181	1	1	1
**GO:0071814**	protein-lipid complex binding	1	21	0.13021	0.30563181	1	1	1
**GO:0030295**	protein kinase activator activity	2	96	0.13266	0.30844201	1	0.88294495	1
**GO:0005164**	tumor necrosis factor receptor binding	1	22	0.13597	0.30844201	1	0.4376939	0.59685532
**GO:0015101**	organic cation transmembrane transporter activity	1	22	0.13597	0.30844201	1	0.4376939	0.59685532

**GO:0016505**	peptidase activator activity involved in apoptotic process	1	22	0.13597	0.30844201	1	0.4376939	1
**GO:0140828**	metal cation:monoatomic cation antiporter activity	1	22	0.13597	0.30844201	1	0.4376939	1
**GO:0019955**	cytokine binding	2	99	0.1394	0.31411467	1	1	1
**GO:0005246**	calcium channel regulator activity	1	23	0.1417	0.31509605	1	0.44761308	0.60626076
**GO:0042910**	xenobiotic transmembrane transporter activity	1	23	0.1417	0.31509605	1	0.44761308	0.60626076
**GO:0001968**	fibronectin binding	1	24	0.14739	0.32560667	1	0.46127611	0.62272275
**GO:0019209**	kinase activator activity	2	103	0.1485	0.32592857	1	0.88294495	1
**GO:0043394**	proteoglycan binding	1	28	0.16977	0.37020813	1	0.52165691	0.70842296
**GO:0000217**	DNA secondary structure binding	1	30	0.18075	0.38666772	1	1	1
**GO:0031369**	translation initiation factor binding	1	30	0.18075	0.38666772	1	0.54547768	0.73606627
**GO:0140662**	ATP-dependent protein folding chaperone	1	30	0.18075	0.38666772	1	0.54547768	0.73606627
**GO:0015026**	coreceptor activity	1	32	0.19158	0.40471275	1	0.56307861	1
**GO:0043028**	cysteine-type endopeptidase regulator activity involved in apoptotic process	1	32	0.19158	0.40471275	1	0.56307861	1
**GO:0030234**	enzyme regulator activity	9	984	0.20108	0.4197042	1	0.98917448	1
**GO:0008289**	lipid binding	6	596	0.20116	0.4197042	1	0.58112889	1
**GO:0004879**	nuclear receptor activity	1	35	0.20756	0.4277761	1	0.58954017	1
**GO:0098531**	ligand-activated transcription factor activity	1	35	0.20756	0.4277761	1	0.58954017	1
**GO:0140318**	protein transporter activity	1	36	0.21282	0.43595855	1	0.599443	0.85634714

**GO:0032813**	tumor necrosis factor receptor superfamily binding	1	37	0.21804	0.44396096	1	0.60878787	1
**GO:0061134**	peptidase regulator activity	2	133	0.21974	0.44474323	1	0.60878787	1
**GO:0015932**	nucleobase-containing compound transmembrane transporter activity	1	41	0.23859	0.48002036	1	1	1
**GO:0004714**	transmembrane receptor protein tyrosine kinase activity	1	42	0.24365	0.4873	1	1	1
**GO:0016504**	peptidase activator activity	1	45	0.25861	0.50819872	1	0.70492081	1
**GO:0042562**	hormone binding	1	45	0.25861	0.50819872	1	0.70492081	1
**GO:1901505**	carbohydrate derivative transmembrane transporter activity	1	45	0.25861	0.50819872	1	1	1
**GO:0003743**	translation initiation factor activity	1	47	0.26843	0.52444705	1	0.72583472	1
**GO:0044183**	protein folding chaperone	1	48	0.27328	0.53085425	1	0.73308444	1
**GO:0004867**	serine-type endopeptidase inhibitor activity	1	50	0.28291	0.54642046	1	0.74878611	1
**GO:0042803**	protein homodimerization activity	5	539	0.28473	0.54681102	1	0.74878611	1
**GO:0061629**	RNA polymerase II-specific DNA-binding transcription factor binding	3	283	0.28819	0.5479943	1	0.74878611	1
**GO:0030695**	GTPase regulator activity	4	412	0.29021	0.5479943	1	0.74878611	1
**GO:0060589**	nucleoside-triphosphatase regulator activity	4	412	0.29021	0.5479943	1	0.74878611	1
**GO:0044877**	protein-containing complex binding	11	1373	0.29638	0.55175714	1	1	1
**GO:0030374**	nuclear receptor coactivator activity	1	53	0.2971	0.55175714	1	0.75503609	1

**GO:0048029**	monosaccharide binding	1	53	0.2971	0.55175714	1	0.75503609	1
**GO:0017022**	myosin binding	1	54	0.30177	0.55736754	1	1	1
**GO:0008080**	N-acetyltransferase activity	1	55	0.30641	0.56286185	1	1	1
**GO:0043621**	protein self-association	1	56	0.31102	0.56654817	1	0.77849424	1
**GO:0005525**	GTP binding	3	298	0.31541	0.56654817	1	0.77849424	1
**GO:0008081**	phosphoric diester hydrolase activity	1	57	0.3156	0.56654817	1	0.77849424	1
**GO:0001618**	virus receptor activity	1	58	0.32015	0.56654817	1	0.77849424	1
**GO:0015079**	potassium ion transmembrane transporter activity	1	58	0.32015	0.56654817	1	0.77849424	1
**GO:0019199**	transmembrane receptor protein kinase activity	1	58	0.32015	0.56654817	1	1	1
**GO:0140272**	exogenous protein binding	1	58	0.32015	0.56654817	1	0.77849424	1
**GO:0001540**	amyloid-beta binding	1	60	0.32916	0.57804969	1	0.78855636	1
**GO:0019001**	guanyl nucleotide binding	3	307	0.33178	0.57804969	1	0.78855636	1
**GO:0032561**	guanyl ribonucleotide binding	3	307	0.33178	0.57804969	1	0.78855636	1
**GO:0030165**	PDZ domain binding	1	61	0.33362	0.57827467	1	0.78855636	1
**GO:0004672**	protein kinase activity	4	448	0.34438	0.59095096	1	1	1
**GO:0019887**	protein kinase regulator activity	2	184	0.34443	0.59095096	1	1	1
**GO:0004222**	metalloendopeptidase activity	1	64	0.34683	0.59206333	1	0.8129826	1
**GO:0001228**	DNA-binding transcription activator activity, RNA polymerase II-specific	3	317	0.34998	0.5934773	1	0.8129826	1
**GO:0005546**	phosphatidylinositol-4,5-bisphosphate binding	1	65	0.35117	0.5934773	1	0.8129826	1
**GO:0001216**	DNA-binding transcription activator activity	3	321	0.35724	0.60073194	1	0.82140898	1

**GO:0042277**	peptide binding	2	192	0.36364	0.60818773	1	0.83047514	1
**GO:0005515**	protein binding	68	10023	0.36825	0.60818773	1	1	1
**GO:0015081**	sodium ion transmembrane transporter activity	1	69	0.36827	0.60818773	1	0.83540443	1
**GO:0140678**	molecular function inhibitor activity	3	329	0.37175	0.60818773	1	1	1
**GO:0004620**	phospholipase activity	1	70	0.37247	0.60818773	1	0.83870697	1
**GO:0016410**	N-acyltransferase activity	1	70	0.37247	0.60818773	1	1	1
**GO:0004197**	cysteine-type endopeptidase activity	1	71	0.37664	0.60996871	1	0.83870697	1
**GO:0003779**	actin binding	3	332	0.37717	0.60996871	1	0.83870697	1
**GO:0050660**	flavin adenine dinucleotide binding	1	72	0.38079	0.61289057	1	0.84122235	1
**GO:0005085**	guanyl-nucleotide exchange factor activity	2	201	0.385	0.61672986	1	0.845	1
**GO:0099106**	ion channel regulator activity	1	74	0.38901	0.62021406	1	0.84829277	1
**GO:0019207**	kinase regulator activity	2	206	0.39674	0.62722692	1	1	1
**GO:0016247**	channel regulator activity	1	76	0.39712	0.62722692	1	0.86042667	1
**GO:0008135**	translation factor activity, RNA binding	1	79	0.40908	0.64013444	1	0.88069452	1
**GO:0016407**	acetyltransferase activity	1	79	0.40908	0.64013444	1	1	1
**GO:0015267**	channel activity	2	215	0.41762	0.64750257	1	0.88294495	1
**GO:0022803**	passive transmembrane transporter activity	2	215	0.41762	0.64750257	1	0.88294495	1
**GO:0004866**	endopeptidase inhibitor activity	1	84	0.4285	0.66073742	1	0.88294495	1
**GO:1902936**	phosphatidylinositol bisphosphate binding	1	85	0.4323	0.66073742	1	0.88294495	1
**GO:0004252**	serine-type endopeptidase activity	1	86	0.43609	0.66073742	1	0.88294495	1
**GO:0016298**	lipase activity	1	86	0.43609	0.66073742	1	0.88294495	1

**GO:0005096**	GTPase activator activity	2	224	0.43813	0.66073742	1	0.88294495	1
**GO:0015297**	antiporter activity	1	87	0.43984	0.66073742	1	0.88294495	1
**GO:0030414**	peptidase inhibitor activity	1	87	0.43984	0.66073742	1	0.88294495	1
**GO:0097367**	carbohydrate derivative binding	12	1691	0.44454	0.66468886	1	1	1
**GO:0015075**	monoatomic ion transmembrane transporter activity	3	371	0.44663	0.66468886	1	0.88294495	1
**GO:0005543**	phospholipid binding	3	372	0.44837	0.66468886	1	0.88294495	1
**GO:0008233**	peptidase activity	3	374	0.45186	0.66691809	1	0.88294495	1
**GO:0004175**	endopeptidase activity	2	231	0.45382	0.66691809	1	0.88294495	1
**GO:0005342**	organic acid transmembrane transporter activity	1	94	0.46546	0.67467314	1	0.88294495	1
**GO:0009055**	electron transfer activity	1	94	0.46546	0.67467314	1	0.88294495	1
**GO:0090079**	translation regulator activity, nucleic acid binding	1	95	0.46903	0.67467314	1	0.88294495	1
**GO:0035091**	phosphatidylinositol binding	2	238	0.46927	0.67467314	1	0.88294495	1
**GO:0019904**	protein domain specific binding	4	533	0.47189	0.67467314	1	0.88294495	1
**GO:0043130**	ubiquitin binding	1	97	0.47609	0.67467314	1	0.88294495	1
**GO:0061135**	endopeptidase regulator activity	1	97	0.47609	0.67467314	1	0.88294495	1
**GO:0003713**	transcription coactivator activity	2	242	0.47798	0.67467314	1	0.88294495	1
**GO:0015078**	proton transmembrane transporter activity	1	98	0.47958	0.67467314	1	0.88294495	1
**GO:0016773**	phosphotransferase activity, alcohol group as acceptor	4	539	0.48063	0.67467314	1	1	1
**GO:0008236**	serine-type peptidase activity	1	99	0.48305	0.67467314	1	0.88294495	1
**GO:0042826**	histone deacetylase binding	1	99	0.48305	0.67467314	1	0.88294495	1

**GO:0017124**	SH3 domain binding	1	101	0.48993	0.67918842	1	0.88294495	1
**GO:0017171**	serine hydrolase activity	1	102	0.49333	0.67918842	1	0.88294495	1
**GO:0043022**	ribosome binding	1	102	0.49333	0.67918842	1	0.88294495	1
**GO:0033218**	amide binding	2	250	0.49514	0.67918842	1	0.88294495	1
**GO:0140297**	DNA-binding transcription factor binding	3	400	0.49633	0.67918842	1	0.88294495	1
**GO:0004713**	protein tyrosine kinase activity	1	109	0.51653	0.70115317	1	1	1
**GO:0051087**	protein-folding chaperone binding	1	109	0.51653	0.70115317	1	0.9140688	1
**GO:0005215**	transporter activity	5	718	0.51927	0.70205304	1	1	1
**GO:0031072**	heat shock protein binding	1	111	0.52296	0.70422502	1	0.9206275	1
**GO:0008237**	metallopeptidase activity	1	113	0.52931	0.70994754	1	0.92697813	1
**GO:0002020**	protease binding	1	114	0.53246	0.71134972	1	0.92768804	1
**GO:0019903**	protein phosphatase binding	1	115	0.53558	0.71139758	1	0.92833867	1
**GO:0032182**	ubiquitin-like protein binding	1	116	0.53868	0.71139758	1	0.92894816	1
**GO:0016301**	kinase activity	4	580	0.53881	0.71139758	1	1	1
**GO:0016922**	nuclear receptor binding	1	117	0.54176	0.71250926	1	0.92951716	1
**GO:0045182**	translation regulator activity	1	123	0.55983	0.73104571	1	0.9467042	1
**GO:0031625**	ubiquitin protein ligase binding	2	282	0.56018	0.73104571	1	0.9467042	1
**GO:0022804**	active transmembrane transporter activity	2	291	0.57738	0.750594	1	1	1
**GO:0005516**	calmodulin binding	1	131	0.58283	0.75477602	1	0.98008229	1
**GO:0044389**	ubiquitin-like protein ligase binding	2	297	0.58858	0.75931313	1	0.98485168	1
**GO:0022857**	transmembrane transporter activity	4	627	0.60139	0.77071682	1	1	1
**GO:0008234**	cysteine-type peptidase activity	1	138	0.60198	0.77071682	1	0.99739824	1

**GO:0008324**	monoatomic cation transmembrane transporter activity	2	307	0.60677	0.77391796	1	1	1
**GO:0022853**	active monoatomic ion transmembrane transporter activity	1	142	0.61253	0.77832759	1	1	1
**GO:0016829**	lyase activity	1	144	0.6177	0.7819573	1	1	1
**GO:1901981**	phosphatidylinositol phosphate binding	1	151	0.63527	0.80119873	1	1	1
**GO:0016887**	ATP hydrolysis activity	2	329	0.64466	0.81001888	1	1	1
**GO:0019902**	phosphatase binding	1	156	0.64733	0.81036126	1	1	1
**GO:0043021**	ribonucleoprotein complex binding	1	159	0.65437	0.81615151	1	1	1
**GO:0008134**	transcription factor binding	3	509	0.66059	0.82088022	1	1	1
**GO:0015291**	secondary active transmembrane transporter activity	1	167	0.67249	0.83260667	1	1	1
**GO:0016747**	acyltransferase activity, transferring groups other than amino-acyl groups	1	172	0.68333	0.84293993	1	1	1
**GO:0016772**	transferase activity, transferring phosphorus-containing groups	4	723	0.71255	0.87095401	1	1	1
**GO:0016491**	oxidoreductase activity	3	551	0.71282	0.87095401	1	1	1
**GO:0005216**	monoatomic ion channel activity	1	187	0.71377	0.87095401	1	1	1
**GO:0017111**	ribonucleoside triphosphate phosphatase activity	3	579	0.74413	0.9047336	1	1	1
**GO:0046873**	metal ion transmembrane transporter activity	1	213	0.75985	0.92053513	1	1	1
**GO:0016462**	pyrophosphatase activity	3	625	0.78972	0.94428601	1	1	1
**GO:0003712**	transcription coregulator activity	2	437	0.79057	0.94428601	1	1	1

**GO:0016817**	hydrolase activity, acting on acid anhydrides	3	626	0.79063	0.94428601	1	1	1
**GO:0016818**	hydrolase activity, acting on acid anhydrides, in phosphorus-containing anhydrides	3	626	0.79063	0.94428601	1	1	1
**GO:0140657**	ATP-dependent activity	2	444	0.79794	0.94966099	1	1	1
**GO:0004857**	enzyme inhibitor activity	1	252	0.81557	0.9672374	1	1	1
**GO:0003924**	GTPase activity	1	255	0.81928	0.96824	1	1	1
**GO:0140096**	catalytic activity, acting on a protein	9	1728	0.82573	0.97136271	1	1	1
**GO:0042578**	phosphoric ester hydrolase activity	1	262	0.82767	0.97136271	1	1	1
**GO:0003729**	mRNA binding	1	272	0.83898	0.9812292	1	1	1
**GO:0000981**	DNA-binding transcription factor activity, RNA polymerase II-specific	4	882	0.84471	0.98452407	1	1	1
**GO:1901702**	salt transmembrane transporter activity	1	281	0.84853	0.9855778	1	1	1
**GO:0022890**	inorganic cation transmembrane transporter activity	1	296	0.86322	0.99579645	1	1	1
**GO:0045296**	cadherin binding	1	296	0.86322	0.99579645	1	1	1
**GO:0003700**	DNA-binding transcription factor activity	4	941	0.87892	1	1	1	1
**GO:0008092**	cytoskeletal protein binding	3	758	0.88547	1	1	1	1
**GO:0005488**	binding	72	11206	0.88744	1	1	1	1
**GO:0036094**	small molecule binding	9	1902	0.90071	1	1	1	1
**GO:0016746**	acyltransferase activity	2	578	0.90112	1	1	1	1
**GO:0046914**	transition metal ion binding	3	796	0.90465	1	1	1	1

**GO:0140110**	transcription regulator activity	6	1376	0.90522	1	1	1	1
**GO:0000978**	RNA polymerase II cis-regulatory region sequence-specific DNA binding	3	802	0.90741	1	1	1	1
**GO:0015318**	inorganic molecular entity transmembrane transporter activity	1	356	0.90918	1	1	1	1
**GO:0004842**	ubiquitin-protein transferase activity	1	361	0.91223	1	1	1	1
**GO:0008270**	zinc ion binding	2	600	0.91248	1	1	1	1
**GO:0016740**	transferase activity	8	1767	0.9145	1	1	1	1
**GO:0000987**	cis-regulatory region sequence-specific DNA binding	3	819	0.91483	1	1	1	1
**GO:0035639**	purine ribonucleoside triphosphate binding	6	1427	0.92264	1	1	1	1
**GO:0019787**	ubiquitin-like protein transferase activity	1	388	0.92706	1	1	1	1
**GO:0016755**	aminoacyltransferase activity	1	400	0.93282	1	1	1	1
**GO:0043168**	anion binding	8	1834	0.93339	1	1	1	1
**GO:0032555**	purine ribonucleotide binding	6	1467	0.93432	1	1	1	1
**GO:0032553**	ribonucleotide binding	6	1479	0.93752	1	1	1	1
**GO:0000166**	nucleotide binding	7	1681	0.94098	1	1	1	1
**GO:1901265**	nucleoside phosphate binding	7	1682	0.94122	1	1	1	1
**GO:0019899**	enzyme binding	7	1706	0.94666	1	1	1	1
**GO:0000977**	RNA polymerase II transcription regulatory region sequence-specific DNA binding	3	930	0.9515	1	1	1	1
**GO:0016787**	hydrolase activity	7	1735	0.95264	1	1	1	1
**GO:0043565**	sequence-specific DNA binding	4	1147	0.9528	1	1	1	1

**GO:0017076**	purine nucleotide binding	6	1554	0.95457	1	1	1	1
**GO:0005524**	ATP binding	4	1159	0.95546	1	1	1	1
**GO:0060090**	molecular adaptor activity	1	486	0.96287	1	1	1	1
**GO:0032559**	adenyl ribonucleotide binding	4	1198	0.9632	1	1	1	1
**GO:0000976**	transcription cis-regulatory region binding	3	1014	0.96892	1	1	1	1
**GO:0001067**	transcription regulatory region nucleic acid binding	3	1016	0.96925	1	1	1	1
**GO:0030554**	adenyl nucleotide binding	4	1284	0.97612	1	1	1	1
**GO:1990837**	sequence-specific double-stranded DNA binding	3	1063	0.97618	1	1	1	1
**GO:0016788**	hydrolase activity, acting on ester bonds	1	554	0.97684	1	1	1	1
**GO:0003690**	double-stranded DNA binding	3	1132	0.98376	1	1	1	1
**GO:0003723**	RNA binding	4	1481	0.99161	1	1	1	1
**GO:0003824**	catalytic activity	17	4193	0.99708	1	1	1	1
**GO:0043167**	ion binding	18	4381	0.99729	1	1	1	1
**GO:0003677**	DNA binding	4	1787	0.99855	1	1	1	1
**GO:0046872**	metal ion binding	10	3064	0.99857	1	1	1	1
**GO:0043169**	cation binding	10	3110	0.99888	1	1	1	1
**GO:0003676**	nucleic acid binding	8	3099	0.99988	1	1	1	1
**GO:1901363**	heterocyclic compound binding	15	4525	0.99992	1	1	1	1
**GO:0097159**	organic cyclic compound binding	15	4572	0.99994	1	1	1	1
**GO:0003674**	molecular_function	79	11942	1	1	1	1	1

Supplemental Table S4: *Inflammatory Infiltrate Assessment*

**Specimen/Treatment**	**Inflammation Presence**	**Type of Inflammation**	**Inflammation Distribution**
401 ETOH	Minimal	Lymphocytic	Perivascular/Superficial Dermal
401 E2	Minimal	Lymphocytic	Superficial dermal/sparse
402 ETOH	No	NA	NA
402 E2	No	NA	NA
403 ETOH	Mild	Lymphocytic	Perifollicular/Superficial Dermal
403 E2	No	NA	NA
404 ETOH	Minimal	Lymphocytic	Superficial dermal/sparse
404 E2	No	NA	NA
405 ETOH	No	NA	NA
405 E2	No	NA	NA

Supplementary Table S5: *Markers for fibroblast subtypes*

**Marker**	**Fibroblast subtype**
SFRP2/PI16	Progenitor-like
CXCL12/CCL19	Inflammatory/immune interacting
COCH/COL11A1	Matrix-associated
CTHRC1/COMP	Activated/myofibroblast-like

Supplementary Figure S2

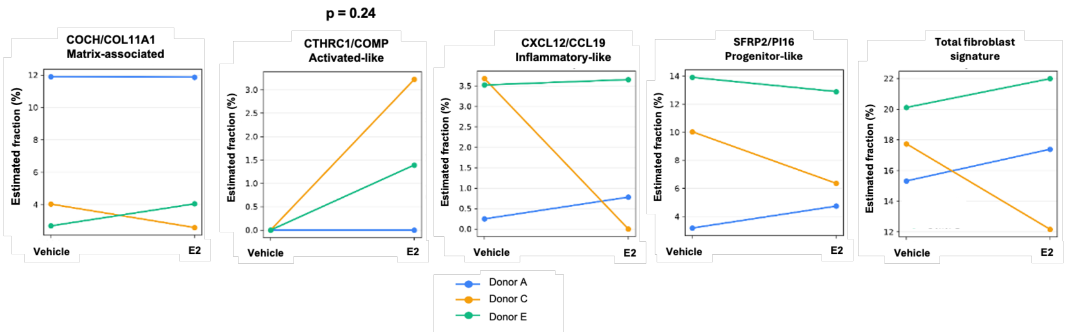

Pilot construct of fibroblast-subtype CIBERSORTx signatures. We used the following markers: COCH/COL11A1, CTHRC1/COMP, CXCL12/CCL19, SFRP2/PI16 in the bulk RNA seq using GSE138669. Increase CTHRC1/COMP signature resulting from E2 treatment in two of three donors (donors C and E vs. donor A), n=3, paired t-test.
